# Decision Support System for Prioritization of Offshore Wind Farm Site by Utilizing Picture Fuzzy Combined Compromise Solution Group Decision Method

**DOI:** 10.3390/e25071081

**Published:** 2023-07-18

**Authors:** Yuan Rong, Liying Yu

**Affiliations:** School of Management, Shanghai University, Shanghai 200444, China; rongyuanry@163.com

**Keywords:** offshore wind farm site, PF set, CoCoSo, PSI, SWARA, information fusion

## Abstract

The selection of offshore wind farm site (OWFS) has important strategic significance for vigorously developing offshore new energy and is deemed as a complicated uncertain multicriteria decision-making (MCDM) process. To further promote offshore wind power energy planning and provide decision support, this paper proposes a hybrid picture fuzzy (PF) combined compromise solution (CoCoSo) technique for prioritization of OWFSs. To begin with, a fresh PF similarity measure is proffered to estimate the importance of experts. Next, the novel operational rules for PF numbers based upon the generalized Dombi norms are defined, and four novel generalized Dombi operators are propounded. Afterward, the PF preference selection index (PSI) method and PF stepwise weights assessment ratio analysis (SWARA) model are propounded to identify the objective and subjective weight of criteria, separately. In addition, the enhanced CoCoSo method is proffered via the similarity measure and new operators for ranking OWFSs with PF information. Lastly, the applicability and feasibility of the propounded PF-PSI-SWARA-CoCoSo method are adopted to ascertain the optimal OWFS. The comparison and sensibility investigations are also carried out to validate the robustness and superiority of our methodology. Results manifest that the developed methodology can offer powerful decision support for departments and managers to evaluate and choose the satisfying OWFSs.

## 1. Introduction

With the continuous deterioration of the global natural environment, chemical energy sources such as oil and coal are becoming increasingly scarce. Exploring renewable and new energy sources has become a focus of attention for countries around the world. As a clean and pollution-free renewable energy source, wind energy is increasingly receiving attention from countries around the world due to its environmentally friendly power generation and huge wind energy reserves. Due to the significant constraints of the power grid, wind resources, and other conditions, the development of offshore wind is approaching saturation. Therefore, offshore wind farms have become an important way to develop clean energy. Offshore wind power generation not only has low operating costs but also has more abundant wind energy resources and a wider geographical area, with considerable development prospects. As an important part of planning for constructing offshore wind farms, the selection of OWFSs involves many factors, such as marine resources, wind power operation, and ship navigation safety [1]. Therefore, how to consider multiple factors, achieve the maximum energy efficiency development of wind farms, and ensure the orderly development of maritime transportation is the key to the site selection of offshore wind farms. Due to the conflicting and inconsistent factors involved in the selection of OWFSs, the process is treated as an MCDM problem in an uncertain environment. In recent years, many scholars have proposed different decision models to provide feasible and effective decision support models for choosing the optimal OWFS [2,3,4,5]. For instance, Gao et al. [6] propounded an MCDGM framework based upon several novel intuitionistic linguistic operators and combined weightdetermination model to rank the considered OWFSs. Wu et al. [7] suggested a new model which combines the weighted operator with stochastic dominance degree within the interval number for ranking the OWFSs. Deveci et al. [8] explored the importance of criteria by LBWA approach by employing the MABAC approach within an interval-valued fuzzy rough setting. Deveci et al. [9] constructed an innovative MCDM model for OWFSs selection using type-2 neutrosophic number. Yu et al. [10] reported an integrated MCDM framework to choose a suitable OWFS based on the proximity indexed value approach using the interval 2-tuple linguistic set and synthetic weight model. In order to think over the psychological behavioral in the course of the decision, Zhao et al. [11] developed a new study for selecting the worthwhile OWFS via the CRITIC method, cumulative prospect theory, and TOPSIS to an ideal solution methods within a spherical fuzzy setting. From the mentioned studies for the selection of OWFSs, we can find that no study merges the combination weight model and CoCoSo method to determine the optimal OWFS(s) within an indeterminacy setting.

The complicated and insufficient cognition of decision problems and the ambiguity of expert behavior cognitive abilities make it difficult to express expert preferences with precise numerical values when providing evaluation opinions based on criteria for established goals. Therefore, how to more accurately offer assessment of the opinion of experts is the key to solving uncertain decision evaluation problems in complex environments. In this regard, fuzzy set (FS) theory was pioneered and extensively utilized to solve the fuzziness of human decision, leading to the in-depth development and application of fuzzy decision [12]. Afterwards, in order to more effectively characterize the uncertain preferences of experts, FS theory was validly extended and obtained uncertain information representation models that can deal with different practical situations, such as intuitionistic FS [13], interval valued intuitionistic FS [14], Pythagorean FS [15], q-rung orthopair FS [16], and spherical FS [17]. The above extensions have been successfully employed in various fields, such as uncertain decision analysis, practical application problem modeling, and so forth [18,19,20,21,22,23,24,25,26]. However, the above extended models based on FS theory only depict the uncertain preferences of experts from the perspectives of membership and nonmembership and cannot effectively depict inconsistent and incomplete information generated in practical problems. In view of this situation, the PF set is originated and regarded as a reasonable and effective tool to more accurately depict the uncertain preferences of experts by considering the grade of membership, nonmembership, and neutral attitude of experts [27]. Afterwards, research on PF set has shown solicitude for attention and has been widely used in the domain of decision theory and method modeling due to its advantages in depicting uncertainty and vague information from multiple perspectives [28,29,30,31,32,33]. In addition, Luo and Zhang [34] introduced an innovative similarity measure for PF sets and proved its superiority and efficiency by the application of pattern recognition. To aggregate PF information for diverse situations effectively, a multitude of aggregation operators are propounded based on different norms and functions. Rong et al. [35] defined a series of prioritized operators based on Archimedean copulas within the PF context to construct an MCDM method. Senapati [36] presented some novel PF Aczel–Alsina operators to build an MCDM approach. Furthermore, to consider the interaction and correlation among the fused data, the PF interactional partitioned Heronian mean operators were developed by Lin et al. [37]. As an important part of MCDM, several novel decision methodologies are extended to the PF setting, such as the REGIME method [38], WASPAS technique [39], COPRAS method [40], CoCoSo approach [41] and so forth. Further, Akram et al. [42] proposed an extended MARCOS technique by a novel information representation model named 2-tuple linguistic q-rung picture fuzzy set. Tian et al. [43] propounded a novel extension of MULTIMOORA method via prospect theory and prioritized operators to select the optimal medical institution. To solve the problems with a large number of experts, Peng et al. [44] brought forward a large-scale group decision method by utilizing the trust-relationship-based social network. Zhao et al. [45] propounded an innovative FMEA approach utilizing the flexible knowledge acquisition with PF information. However, there is no study that introduces the generalized Dombi operator [46] to the PF setting for decision analysis.

The recent literature has shown that a large number of classic decision models have been extended to determine the sorting of schemes under PF environments for resolving practical problems. Each of the mentioned PF methods possesses its own advantages and disadvantages in the process of unfolding complicated decision analysis. The CoCoSo method is a fresh approach to attain the priority ranking of solutions by means of weighted sum, weighted product models, and three fusion strategies from different perspectives [47]. Its main advantages are that it is based on the combination perspective and the compromise perspective to determine the ranking of schemes, and it possesses the strengths of evading the compensation problem and realizing the internal balance of the ultimate utility, as well as relatively low computational complexity. Owing to these merits of the CoCoSo technique in decision fields, it has been generalized to diverse uncertain circumstances for enriching the development of the CoCoSo methodology [48,49,50,51]. Further, Deveci et al. [52] propounded an innovative CoCoSo method via power Heronian operator to consider the correlation interactive of autonomous vehicles criteria with fuzzy information. Chen et al. [53] presented an occupational health and safety risk assessment model based upon an expanded CoCoSo method within Fermatean fuzzy linguistic circumstance. Lai et al. [54] proposed a novel extension of the CoCoSo method by using the hesitant Fermatean FS and used the approach to evaluate the blockchain platform. Bouraima et al. [55] introduced a novel decision framework by merging SWARA and CoCoSo models within interval rough numbers for the evaluation of railway system alternatives. From all the mentioned extended versions of the CoCoSo method, it has been found that CoCoSo model fails to have been employed based on the generalized Dombi operator under PF circumstance.

With the aid of the aforementioned discussion, this study aims to propose some novel generalized Dombi operators under the PF setting and then build an MCGDM methodology via the enhanced CoCoSo technique that uses the defined operators. As a consequence, the motivations are outlined below:

♠ The existing works have provided different MCDM methodologies to select a suitable OWFS, but there is no research that offers a comprehensive decision framework to handle indeterminacy and vagueness of assessment from the PF perspective.
♠ The extant PF decision approaches have introduced some criteria weight computational models, but few work presents the synthetic weight determination method to obtain weight information from the two angles of subjectivity and objectivity.
♠ The generalized Dombi operation is not only a generalized form of the extant operators but also possess stronger flexibility via two parameters during the course of information fusion. However, it fails to be extended to the PF circumstance.
♠ The classical CoCoSo technique acquires the weighted sum and weighted product measures by the basic weighted average operator, which is short on flexibility and robustness. Hence, it is imperative to strengthen the original CoCoSo model by some novel operators.

By means of the aforementioned motivations, the target of the current article is to construct an integrated framework via uniting the CoCoSo model, generalized Dombi operator, and synthetic weight determination model under the PF circumstance. In the method, a novel model is formulated via the improved PF-PSI and PF-SWARA methods to estimate the weight of criteria and an enhanced CoCoSo method using the novel generalized Dombi operators is advanced to rank the considered OWFSs. Driven by the mentioned targets, the main novelties of this article are summarized as follows:

¶ We bring forward an enhanced CoCoSo method by novel integration operator and similarity measure to acquire optimal OWFS under the PF context.
¶ In the designed framework, the PF-PSI method and PF-SWARA approach are, respectively, developed to ascertain the objective and subjective weight of criteria for evaluating the OWFSs, which strengthens the accurateness of the criteria weight.
¶ Inspired by the merits of generalized Dombi norms, the PF generalized Dombi operational rules are defined, and some novel PF generalized Dombi operators like PFGDWA, PFGDWA, PFGDGWA, and PFGDOWG are propounded to fuse PF information.
¶ A novel PF similarity measure is formulated to evaluate the weight of experts by using a similarity-based approach.
¶ The feasibility and effectiveness of the developed PF-PSI-SWARA-CoCoSo decision framework is demonstrated through a case study on OWFSs selection and a contrastive analysis with other methods.
¶ Sensitivity and contrastive discussions are carried out to analyze the robustness and advantages of the proffered MCGDM approach.

The rest of the sections of this paper are as follows. Section 2 succinctly retrospects some fundamental concepts of PF sets. Section 3 propounds a novel PF similarity measure and gives its related proof. Section 4 defines the generalized Dombi operations and some novel PF generalized Dombi operators. A hybrid PF-MCGDM method called the PF-PSI-SWARA-CoCoSo methodology is constructed in Section 5. In Section 6, the PF-PSI-SWARA-CoCoSo approach is adopted to ascertain the optimal OWFS and further show its feasibility, and a contrastive analysis is also implemented to discuss the strengths of the developed methodology. Section 7 gives several conclusions and future directions.

## 2. Preliminary Notions

Several necessary preliminaries of this paper, including the basic notions of PF set and generalized Dombi operations, are retrospected in this part.

### 2.1. PF Set

**Definition** **1** ([27])**.** *A PF set $\tilde{A}$ over the universe of discourse X is expressed as*

(1) $$\begin{array}{c} \tilde{A}=\left\{\langle \chi ,{\tilde{\mu }}_{\tilde{A}}\left(\chi \right),{\tilde{\eta }}_{\tilde{A}}\left(\chi \right),{\tilde{\xi }}_{\tilde{A}}\left(\chi \right)\rangle |\chi \in X\right\} \end{array}$$

*in which ${\tilde{\mu }}_{\tilde{A}}\left(\chi \right)$ signifies the positive membership function with ${\tilde{\mu }}_{\tilde{A}}:X\to \left[0,1\right]$, $\chi \in X\to {\tilde{\mu }}_{\tilde{A}}\left(\chi \right)\in \left[0,1\right]$, ${\tilde{\eta }}_{\tilde{A}}\left(\chi \right)$ signifies the neutral membership function with ${\tilde{\eta }}_{\tilde{A}}:X\to \left[0,1\right]$, $\chi \in X\to {\tilde{\eta }}_{\tilde{A}}\left(\chi \right)\in \left[0,1\right]$, and $\xi_{\tilde{A}}\left(\chi \right)$ signifies the negative membership function with $\xi_{\tilde{A}}$: X→[0,1], $\chi \in X\to {\tilde{\xi }}_{\tilde{A}}\left(x\right)\in \left[0,1\right]$. The above membership functions comply with the condition $0\leq {\tilde{\mu }}_{\tilde{A}}\left(\chi \right)+{\tilde{\eta }}_{\tilde{A}}\left(\chi \right)+{\tilde{\xi }}_{\tilde{A}}\left(\chi \right)\leq 1$ for all χ∈X. Meanwhile, the refusal degree of the PF set $\tilde{A}$ can be expounded as ${\tilde{\pi }}_{\tilde{A}}\left(\chi \right)=1-{\tilde{\mu }}_{\tilde{A}}\left(\chi \right)-{\tilde{\eta }}_{\tilde{A}}\left(\chi \right)-{\tilde{\xi }}_{\tilde{A}}\left(\chi \right)$ for all χ∈X. Also, $0\leq {\tilde{\pi }}_{\tilde{A}}\left(\chi \right)\leq 1$ for all χ∈X. For simplicity of calculation, $\tilde{\alpha }=\left({\tilde{\mu }}_{\tilde{\alpha }},{\tilde{\eta }}_{\tilde{\alpha }},{\tilde{\xi }}_{\tilde{\alpha }}\right)$ indicates a PF number.*

**Definition** **2** ([29])**.** *Let $\tilde{\alpha }=\left({\tilde{\mu }}_{\tilde{\alpha }},{\tilde{\eta }}_{\tilde{\alpha }},{\tilde{\xi }}_{\tilde{\alpha }}\right)$, ${\tilde{\alpha }}_{1}=\left({\tilde{\mu }}_{{\tilde{\alpha }}_{1}},{\tilde{\eta }}_{{\tilde{\alpha }}_{1}},{\tilde{\xi }}_{{\tilde{\alpha }}_{1}}\right)$, and ${\tilde{\alpha }}_{2}=\left({\tilde{\mu }}_{{\tilde{\alpha }}_{2}},{\tilde{\eta }}_{{\tilde{\alpha }}_{2}},{\tilde{\xi }}_{{\tilde{\alpha }}_{2}}\right)$ be three PF numbers. Then, the operational rules of PF numbers are defined as follows:*

(2) $$\begin{array}{c} (1)\;{\tilde{\alpha }}_{1}\cup {\tilde{\alpha }}_{2}=\left(\max\left({\tilde{\mu }}_{{\tilde{\alpha }}_{1}},{\tilde{\mu }}_{{\tilde{\alpha }}_{2}}\right),\min\left({\tilde{\eta }}_{{\tilde{\alpha }}_{1}},{\tilde{\eta }}_{{\tilde{\alpha }}_{2}}\right),\min\left({\tilde{\xi }}_{{\tilde{\alpha }}_{1}},{\tilde{\xi }}_{{\tilde{\alpha }}_{2}}\right)\right); \end{array}$$

(3) $$\begin{array}{c} (2)\;{\tilde{\alpha }}_{1}\cap {\tilde{\alpha }}_{2}=\left(\min\left({\tilde{\mu }}_{{\tilde{\alpha }}_{1}},{\tilde{\mu }}_{{\tilde{\alpha }}_{2}}\right),\max\left({\tilde{\eta }}_{{\tilde{\alpha }}_{1}},{\tilde{\eta }}_{{\tilde{\alpha }}_{2}}\right),\max\left({\tilde{\xi }}_{{\tilde{\alpha }}_{1}},{\tilde{\xi }}_{{\tilde{\alpha }}_{2}}\right)\right); \end{array}$$

(4) $$\begin{array}{c} (3)\;{\tilde{\alpha }}_{1}⊕{\tilde{\alpha }}_{2}=\left({\tilde{\mu }}_{{\tilde{\alpha }}_{1}}+{\tilde{\mu }}_{{\tilde{\alpha }}_{2}}-{\tilde{\mu }}_{{\tilde{\alpha }}_{1}}{\tilde{\mu }}_{{\tilde{\alpha }}_{2}},{\tilde{\eta }}_{{\tilde{\alpha }}_{1}}{\tilde{\eta }}_{{\tilde{\alpha }}_{2}},{\tilde{\xi }}_{{\tilde{\alpha }}_{1}}{\tilde{\xi }}_{{\tilde{\alpha }}_{2}}\right); \end{array}\;\;\;\;\;\;$$

(5) $$\begin{array}{c} (4)\;{\tilde{\alpha }}_{1}⊗{\tilde{\alpha }}_{2}=\left({\tilde{\mu }}_{{\tilde{\alpha }}_{1}}{\tilde{\mu }}_{{\tilde{\alpha }}_{2}},{\tilde{\eta }}_{{\tilde{\alpha }}_{1}}+{\tilde{\eta }}_{{\tilde{\alpha }}_{2}}-{\tilde{\eta }}_{{\tilde{\alpha }}_{1}}{\tilde{\eta }}_{{\tilde{\alpha }}_{2}},{\tilde{\xi }}_{{\tilde{\alpha }}_{1}}+{\tilde{\xi }}_{{\tilde{\alpha }}_{2}}-{\tilde{\xi }}_{{\tilde{\alpha }}_{1}}{\tilde{\xi }}_{{\tilde{\alpha }}_{2}}\right); \end{array}\;$$

(6) $$\begin{array}{c} (5)\;\lambda \tilde{\alpha }=\left(1-{\left(1-{\tilde{\mu }}_{\tilde{\alpha }}\right)}^{\lambda },{\left({\tilde{\eta }}_{\tilde{\alpha }}\right)}^{\lambda },{\left({\tilde{\xi }}_{\tilde{\alpha }}\right)}^{\lambda }\right),\lambda >0; \end{array}\;\;\;\;\;\;\;$$

(7) $$\begin{array}{c} (6)\;{\tilde{\alpha }}^{\lambda }=\left({\left({\tilde{\mu }}_{\tilde{\alpha }}\right)}^{\lambda },1-{\left(1-{\tilde{\eta }}_{\tilde{\alpha }}\right)}^{\lambda },1-{\left(1-{\tilde{\xi }}_{\tilde{\alpha }}\right)}^{\lambda }\right),\lambda >0. \end{array}\;\;\;\;$$

In order to attain the order relation of two PF numbers, the score function $SF\left(\tilde{\alpha }\right)$ and accuracy function $AF\left(\tilde{\alpha }\right)$ of a PF number $\tilde{\alpha }=\left({\tilde{\mu }}_{\tilde{\alpha }},{\tilde{\eta }}_{\tilde{\alpha }},{\tilde{\xi }}_{\tilde{\alpha }}\right)$ are defined as $SF\left(\tilde{\alpha }\right)={\tilde{\mu }}_{\tilde{\alpha }}-{\tilde{\xi }}_{\tilde{\alpha }}$ and $AF\left(\tilde{\alpha }\right)={\tilde{\mu }}_{\tilde{\alpha }}+{\tilde{\eta }}_{\tilde{\alpha }}+{\tilde{\xi }}_{\tilde{\alpha }}$, respectively [29]. However, the mentioned score and accuracy function fail to finish the comparison of two PF numbers in some situations. Accordingly, a novel score function is originated to conquer the defect, which is defined as

**Definition** **3** ([35])**.** *Given a PF number $\tilde{\alpha }=\left({\tilde{\mu }}_{\tilde{\alpha }},{\tilde{\eta }}_{\tilde{\alpha }},{\tilde{\xi }}_{\tilde{\alpha }}\right)$, the score of $\tilde{\alpha }$ is defined as follows:*

(8) $$\begin{array}{cc} \; & \tilde{SF}\left(\tilde{\alpha }\right)=\frac{e^{{\tilde{\mu }}_{\tilde{\alpha }}-{\tilde{\eta }}_{\tilde{\alpha }}-{\tilde{\xi }}_{\tilde{\alpha }}}}{2-{\tilde{\mu }}_{\tilde{\alpha }}-{\tilde{\eta }}_{\tilde{\alpha }}-{\tilde{\xi }}_{\tilde{\alpha }}},\;\tilde{SF}\left(\tilde{\alpha }\right)\in \left[e^{-1},e\right]. \end{array}$$

**Definition** **4** ([35])**.** *Given two PF numbers ${\tilde{\alpha }}_{1}=\left({\tilde{\mu }}_{{\tilde{\alpha }}_{1}},{\tilde{\eta }}_{{\tilde{\alpha }}_{1}},{\tilde{\xi }}_{{\tilde{\alpha }}_{1}}\right)$ and ${\tilde{\alpha }}_{2}=\left({\tilde{\mu }}_{{\tilde{\alpha }}_{2}},{\tilde{\eta }}_{{\tilde{\alpha }}_{2}},{\tilde{\xi }}_{{\tilde{\alpha }}_{2}}\right)$, the comparison rules are described as follows:*

- *If $\tilde{SF}\left({\tilde{\alpha }}_{1}\right)<\tilde{SF}\left({\tilde{\alpha }}_{2}\right)$, then ${\tilde{\alpha }}_{1}≺{\tilde{\alpha }}_{2}$;*
- *If $\tilde{SF}\left({\tilde{\alpha }}_{1}\right)=\tilde{SF}\left({\tilde{\alpha }}_{2}\right)$, then:*
  - *If $AF\left({\tilde{\alpha }}_{1}\right)>AF\left({\tilde{\alpha }}_{2}\right)$, then ${\tilde{\alpha }}_{1}≻{\tilde{\alpha }}_{2}$;*
  - *If $AF\left({\tilde{\alpha }}_{1}\right)=AF\left({\tilde{\alpha }}_{2}\right)$, then ${\tilde{\alpha }}_{1}∼{\tilde{\alpha }}_{2}$.*

**Definition** **5** ([28])**.** *Let $\tilde{A}$,$\tilde{B}$ and $\tilde{C}$ be three PF sets. Then, a PF similarity measure SM is a mapping that meets the following properties:*

*(P1) $0\leq SM\left(\tilde{A},\tilde{B}\right)\leq 1$;*

*(P2) $SM\left(\tilde{A},\tilde{B}\right)=SM\left(\tilde{B},\tilde{A}\right)$;*

*(P3) $SM\left(\tilde{A},\tilde{B}\right)=1\Leftrightarrow \tilde{A}=\tilde{B}$;*

*(P4) If $\tilde{A}\subseteq \tilde{B}\subseteq \tilde{A}$, then $SM\left(\tilde{A},\tilde{C}\right)\leq SM\left(\tilde{A},\tilde{B}\right)$ and $SM\left(\tilde{A},\tilde{C}\right)\leq SM\left(\tilde{B},\tilde{C}\right)$.*

### 2.2. Generalized Dombi Operations

**Definition** **6** ([46])**.** *The generalized Dombi operators originated by Dombi are defined as below:*

(9) $$\begin{array}{cc} \; & GD_{t}^{s}\left(x_{1},x_{2}\right)={\left(1+{\left(\frac{1}{t}\left(\prod_{j=1}^{2}\Psi_{t}^{s}\left(x_{j}\right)-1\right)\right)}^{\frac{1}{s}}\right)}^{-1}, \end{array}$$

(10) $$\begin{array}{cc} \; & {\bar{GD}}_{t}^{s}\left(x_{1},x_{2}\right)={\left(1+{\left(\frac{1}{t}\left(\prod_{j=1}^{2}\Phi_{t}^{s}\left(x_{j}\right)-1\right)\right)}^{-\frac{1}{s}}\right)}^{-1}, \end{array}$$

*where $\Psi_{t}^{s}\left(x_{j}\right)=1+t{\left(\frac{1-x_{j}}{x_{j}}\right)}^{s}$, $\Phi_{t}^{s}\left(x_{j}\right)=1+t{\left(\frac{x_{j}}{1-x_{j}}\right)}^{s}$, $\left(x_{j}\in \left(0,1\right),j=1,2\right)$, and t>0.*

The generalized Dombi operations possess elegant properties, including generalization and flexibility, which can reduce to other existing operations and highlight the flexible control capability via the parameters.

## 3. A Novel Similarity Measure

The similarity measure is an important branch of information measure in FS theory, which can effectively distinguish the similarity between two objectives. Numerous research achievements on FS are propounded to pattern recognition, cluster analysis, and decision analysis [28,34] Furthermore, as an efficiency extension of fuzzy sets, several PF similarity measures are developed for enriching the information measure theory and utilized in practical problems. In this part, a novel PF similarity measure is put forward as the basic for the establishment of decision methodology.

**Definition** **7.** *Let $\tilde{A}$ and $\tilde{B}$ be two PF sets. Then, a novel PF similarity measure $SM\left(\tilde{A},\tilde{B}\right)$ is presented as*

(11) $$\begin{array}{c} SM\left(\tilde{A},\tilde{B}\right)=\frac{\sum_{q=1}^{n}\left[\min\left\{{\tilde{\mu }}_{\tilde{A}}\left(x_{q}\right),{\tilde{\mu }}_{\tilde{B}}\left(x_{q}\right)\right\}+\min\left\{\left(1-{\tilde{\eta }}_{\tilde{A}}\left(x_{q}\right)\right),\left(1-{\tilde{\eta }}_{\tilde{B}}\left(x_{q}\right)\right)\right\}+\min\left\{\left(1-{\tilde{\xi }}_{\tilde{A}}\left(x_{q}\right)\right),\left(1-{\tilde{\xi }}_{\tilde{B}}\left(x_{q}\right)\right)\right\}\right]}{\sum_{q=1}^{n}\left[\max\left\{{\tilde{\mu }}_{\tilde{A}}\left(x_{q}\right),{\tilde{\mu }}_{\tilde{B}}\left(x_{q}\right)\right\}+\max\left\{\left(1-{\tilde{\eta }}_{\tilde{A}}\left(x_{q}\right)\right),\left(1-{\tilde{\eta }}_{\tilde{B}}\left(x_{q}\right)\right)\right\}+\max\left\{\left(1-{\tilde{\xi }}_{\tilde{A}}\left(x_{q}\right)\right),\left(1-{\tilde{\xi }}_{\tilde{B}}\left(x_{q}\right)\right)\right\}\right]}. \end{array}$$

**Theorem** **1.** *The mapping $SM\left(\tilde{A},\tilde{B}\right)$ is a PF similarity measure.*

**Proof.**  The proof of Theorem 1 is displayed in Appendix A. □

## 4. Some Novel PF Generalized Dombi Operators

In this section, we first define the generalized Dombi operations for PF numbers. Then, we propound several novel PF generalized Dombi weighted averaging and geometric operators, as well as the corresponding elegant properties and special instances also being investigated.

### 4.1. Generalized Dombi Operations for PF Numbers

Herein, the PF generalized Dombi operational laws are presented, and their fundamental properties are also discussed.

**Definition** **8.** *Let $\tilde{\alpha }=\left({\tilde{\mu }}_{\tilde{\alpha }},{\tilde{\eta }}_{\tilde{\alpha }},{\tilde{\xi }}_{\tilde{\alpha }}\right)$, ${\tilde{\alpha }}_{q}=\left({\tilde{\mu }}_{{\tilde{\alpha }}_{q}},{\tilde{\eta }}_{{\tilde{\alpha }}_{q}},{\tilde{\xi }}_{{\tilde{\alpha }}_{q}}\right)\left(q=1,2\right)$ be three PF numbers. Then, the PF generalized Dombi operational laws on them are defined as follows:*

(12) $$\begin{array}{cc} \; & \left(1\right)\;{\tilde{\alpha }}_{1}\tilde{⊕}{\tilde{\alpha }}_{2}=\left({\left(1+{\left(\frac{1}{t}\left(\prod_{q=1}^{2}\Phi_{t}^{s}\left({\tilde{\mu }}_{{\tilde{\alpha }}_{q}}\right)-1\right)\right)}^{-\frac{1}{s}}\right)}^{-1},{\left(1+{\left(\frac{1}{t}\left(\prod_{q=1}^{2}\Psi_{t}^{s}\left({\tilde{\eta }}_{{\tilde{\alpha }}_{q}}\right)-1\right)\right)}^{\frac{1}{s}}\right)}^{-1},{\left(1+{\left(\frac{1}{t}\left(\prod_{q=1}^{2}\Psi_{t}^{s}\left({\tilde{\xi }}_{{\tilde{\alpha }}_{q}}\right)-1\right)\right)}^{\frac{1}{s}}\right)}^{-1}\right); \end{array}$$

(13) $$\begin{array}{cc} \; & \left(2\right)\;{\tilde{\alpha }}_{1}\tilde{⊗}{\tilde{\alpha }}_{2}=\left({\left(1+{\left(\frac{1}{t}\left(\prod_{q=1}^{2}\Psi_{t}^{s}\left({\tilde{\mu }}_{{\tilde{\alpha }}_{q}}\right)-1\right)\right)}^{\frac{1}{s}}\right)}^{-1},{\left(1+{\left(\frac{1}{t}\left(\prod_{q=1}^{2}\Phi_{t}^{s}\left({\tilde{\eta }}_{{\tilde{\alpha }}_{q}}\right)-1\right)\right)}^{-\frac{1}{s}}\right)}^{-1},{\left(1+{\left(\frac{1}{t}\left(\prod_{q=1}^{2}\Phi_{t}^{s}\left({\tilde{\xi }}_{{\tilde{\alpha }}_{q}}\right)-1\right)\right)}^{-\frac{1}{s}}\right)}^{-1}\right); \end{array}$$

(14) $$\begin{array}{cc} \; & \left(3\right)\;\lambda \tilde{\alpha }=\left({\left(1+{\left(\frac{1}{t}\left({\left(\Phi_{t}^{s}\left({\tilde{\mu }}_{\tilde{\alpha }}\right)\right)}^{\lambda }-1\right)\right)}^{-\frac{1}{s}}\right)}^{-1},{\left(1+{\left(\frac{1}{t}\left({\left(\Psi_{t}^{s}\left({\tilde{\eta }}_{\tilde{\alpha }}\right)\right)}^{\lambda }-1\right)\right)}^{\frac{1}{s}}\right)}^{-1},{\left(1+{\left(\frac{1}{t}\left({\left(\Psi_{t}^{s}\left({\tilde{\xi }}_{\tilde{\alpha }}\right)\right)}^{\lambda }-1\right)\right)}^{\frac{1}{s}}\right)}^{-1}\right),\lambda >0; \end{array}$$

(15) $$\begin{array}{cc} \; & \left(4\right)\;{\tilde{\alpha }}^{\lambda }=\left({\left(1+{\left(\frac{1}{t}\left({\left(\Psi_{t}^{s}\left({\tilde{\mu }}_{\tilde{\alpha }}\right)\right)}^{\lambda }-1\right)\right)}^{\frac{1}{s}}\right)}^{-1},{\left(1+{\left(\frac{1}{t}\left({\left(\Phi_{t}^{s}\left({\tilde{\eta }}_{\tilde{\alpha }}\right)\right)}^{\lambda }-1\right)\right)}^{-\frac{1}{s}}\right)}^{-1},{\left(1+{\left(\frac{1}{t}\left({\left(\Phi_{t}^{s}\left({\tilde{\xi }}_{\tilde{\alpha }}\right)\right)}^{\lambda }-1\right)\right)}^{-\frac{1}{s}}\right)}^{-1}\right),\lambda >0. \end{array}$$

**Theorem** **2.** *Suppose that ${\tilde{\alpha }}_{q}=\left({\tilde{\mu }}_{{\tilde{\alpha }}_{q}},{\tilde{\eta }}_{{\tilde{\alpha }}_{q}},{\tilde{\xi }}_{{\tilde{\alpha }}_{q}}\right)\left(q=1,2\right)$ is two PF numbers and $\lambda ,\lambda_{1},\lambda_{2}>0$. Then, one has*

$$\begin{array}{cc} (1)\; & {\tilde{\alpha }}_{1}\tilde{⊕}{\tilde{\alpha }}_{2}={\tilde{\alpha }}_{2}\tilde{⊕}{\tilde{\alpha }}_{1};\\ (2)\; & {\tilde{\alpha }}_{1}\tilde{⊗}{\tilde{\alpha }}_{2}={\tilde{\alpha }}_{2}\tilde{⊗}{\tilde{\alpha }}_{1};\\ (3)\; & \lambda \cdot \left({\tilde{\alpha }}_{1}\tilde{⊕}{\tilde{\alpha }}_{2}\right)=\left(\lambda \cdot {\tilde{\alpha }}_{1}\right)\tilde{⊕}\left(\lambda {\tilde{\alpha }}_{2}\right);\\ (4)\; & \left(\lambda_{1}\cdot {\tilde{\alpha }}_{1}\right)\tilde{⊕}\left(\lambda_{2}\cdot {\tilde{\alpha }}_{1}\right)=\left(\lambda_{1}+\lambda_{2}\right)\cdot {\tilde{\alpha }}_{1};\\ (5)\; & {\left({\tilde{\alpha }}_{1}\tilde{⊗}{\tilde{\alpha }}_{2}\right)}^{\lambda }=\left({\tilde{\alpha }}_{1}^{\lambda }\right)\tilde{⊗}\left({\tilde{\alpha }}_{2}^{\lambda }\right);\\ (6)\; & \left({\tilde{\alpha }}_{1}^{\lambda_{1}}\right)\tilde{⊗}\left({\tilde{\alpha }}_{1}^{\lambda_{2}}\right)={\tilde{\alpha }}_{1}^{\left(\lambda_{1}+\lambda_{2}\right)}. \end{array}$$

**Proof.**  It can be proved easily by the Definition 8. □

### 4.2. Some PF Generalized Dombi Weighted Averaging Operators

This subsection propounds the PF generalized Dombi weighted averaging (PFGDWA) operator, the PF generalized Dombi ordered weighted averaging (PFGDOWA) operator, and their elegant properties.

**Definition** **9.** *Suppose that ${\tilde{\alpha }}_{q}=\left({\tilde{\mu }}_{{\tilde{\alpha }}_{q}},{\tilde{\eta }}_{{\tilde{\alpha }}_{q}},{\tilde{\xi }}_{{\tilde{\alpha }}_{q}}\right)\left(q=1\left(1\right)n\right)$ is a family of PF numbers and $\delta ={\left(\delta_{1},\delta_{2},\cdots ,\delta_{n}\right)}^{T}$ is the weight vector of ${\tilde{\alpha }}_{q}$, with $\delta_{q}>0$ and $\sum_{q=1}^{n}\delta_{q}=1$. Then, the PFGDWA operator is a mapping from $\Omega^{n}$ to Ω, expressed as*

(16) $$\begin{array}{c} PFGDWA\left({\tilde{\alpha }}_{1},{\tilde{\alpha }}_{2},\cdots ,{\tilde{\alpha }}_{n}\right)=\overset{n}{\underset{q=1}{\tilde{⊕}}}\left(\delta_{q}{\tilde{\alpha }}_{q}\right). \end{array}$$

In light of the Definition 9, the following theorems are developed.

**Theorem** **3.** *The fused value $PFGDWA\left({\tilde{\alpha }}_{1},{\tilde{\alpha }}_{2},\cdots ,{\tilde{\alpha }}_{n}\right)$ is still a PF number, portrayed as*

(17) $$\begin{array}{cc} \; & PFGDWA\left({\tilde{\alpha }}_{1},{\tilde{\alpha }}_{2},\cdots ,{\tilde{\alpha }}_{n}\right)=\overset{n}{\underset{q=1}{\tilde{⊕}}}\left(\delta_{q}{\tilde{\alpha }}_{q}\right)\\ \; & \left({\left(1+{\left(\frac{1}{t}\left(\prod_{q=1}^{n}{\left(\Phi_{t}^{s}\left({\tilde{\mu }}_{{\tilde{\alpha }}_{q}}\right)\right)}^{\delta_{q}}-1\right)\right)}^{-\frac{1}{s}}\right)}^{-1},{\left(1+{\left(\frac{1}{t}\left(\prod_{q=1}^{n}{\left(\Psi_{t}^{s}\left({\tilde{\eta }}_{{\tilde{\alpha }}_{q}}\right)\right)}^{\delta_{q}}-1\right)\right)}^{\frac{1}{s}}\right)}^{-1},{\left(1+{\left(\frac{1}{t}\left(\prod_{q=1}^{n}{\left(\Psi_{t}^{s}\left({\tilde{\xi }}_{{\tilde{\alpha }}_{q}}\right)\right)}^{\delta_{q}}-1\right)\right)}^{\frac{1}{s}}\right)}^{-1}\right). \end{array}$$

**Proof.**  The proof of Theorem 3 is displayed in Appendix B. □

In what follows, we discuss several special cases of the PFGDWA operator.

**Case** **1.** *If s=1 and t=1, then the PFGDWA operator is degenerated into the PF weighted averaging (PFWA) operator.*

$$\begin{array}{c} PFWA\left({\tilde{\alpha }}_{1},{\tilde{\alpha }}_{2},\cdots ,{\tilde{\alpha }}_{n}\right)=\left(1-\prod_{q=1}^{n}{\left(1-{\tilde{\mu }}_{{\tilde{\alpha }}_{q}}\right)}^{\delta_{q}},\prod_{q=1}^{n}{\left({\tilde{\eta }}_{{\tilde{\alpha }}_{q}}\right)}^{\delta_{q}},\prod_{q=1}^{n}{\left({\tilde{\xi }}_{{\tilde{\alpha }}_{q}}\right)}^{\delta_{q}}\right) \end{array}$$

**Case** **2.** *If s=1 and t=2, then the PFGDWA operator is degenerated into the PF Einstein weighted averaging (PFWA) operator.*

$$\begin{array}{c} PFEWA\left({\tilde{\alpha }}_{1},{\tilde{\alpha }}_{2},\cdots ,{\tilde{\alpha }}_{n}\right)=\left(\frac{\prod_{q=1}^{n}{\left(1+{\tilde{\mu }}_{{\tilde{\alpha }}_{q}}\right)}^{\delta_{q}}-\prod_{q=1}^{n}{\left(1-{\tilde{\mu }}_{{\tilde{\alpha }}_{q}}\right)}^{\delta_{q}}}{\prod_{q=1}^{n}{\left(1+{\tilde{\mu }}_{{\tilde{\alpha }}_{q}}\right)}^{\delta_{q}}+\prod_{q=1}^{n}{\left(1-{\tilde{\mu }}_{{\tilde{\alpha }}_{q}}\right)}^{\delta_{q}}},\frac{2\prod_{q=1}^{n}{\left({\tilde{\eta }}_{{\tilde{\alpha }}_{q}}\right)}^{\delta_{q}}}{\prod_{q=1}^{n}{\left(2-{\tilde{\eta }}_{{\tilde{\alpha }}_{q}}\right)}^{\delta_{q}}+\prod_{q=1}^{n}{\left({\tilde{\eta }}_{{\tilde{\alpha }}_{q}}\right)}^{\delta_{q}}},\frac{2\prod_{q=1}^{n}{\left({\tilde{\xi }}_{{\tilde{\alpha }}_{q}}\right)}^{\delta_{q}}}{\prod_{q=1}^{n}{\left(2-{\tilde{\xi }}_{{\tilde{\alpha }}_{q}}\right)}^{\delta_{q}}+\prod_{q=1}^{n}{\left({\tilde{\xi }}_{{\tilde{\alpha }}_{q}}\right)}^{\delta_{q}}},\right) \end{array}$$

**Case** **3.** *If s=1, then the PFGDWA operator is degenerated into the PF Hamacher weighted averaging (PFHWA) operator.*

$$\begin{array}{cc} \; & PFHWA\left({\tilde{\alpha }}_{1},{\tilde{\alpha }}_{2},\cdots ,{\tilde{\alpha }}_{n}\right)=\\ \; & \left(\frac{\prod_{q=1}^{n}{\left(1+\left(t-1\right){\tilde{\mu }}_{{\tilde{\alpha }}_{q}}\right)}^{\delta_{q}}-\prod_{q=1}^{n}{\left(1-{\tilde{\mu }}_{{\tilde{\alpha }}_{q}}\right)}^{\delta_{q}}}{\prod_{q=1}^{n}{\left(1+\left(t-1\right){\tilde{\mu }}_{{\tilde{\alpha }}_{q}}\right)}^{\delta_{q}}+\left(t-1\right)\prod_{q=1}^{n}{\left(1-{\tilde{\mu }}_{{\tilde{\alpha }}_{q}}\right)}^{\delta_{q}}},\frac{t\prod_{q=1}^{n}{\left({\tilde{\eta }}_{{\tilde{\alpha }}_{q}}\right)}^{\delta_{q}}}{\prod_{q=1}^{n}{\left(1+\left(t-1\right)\left(1-{\tilde{\eta }}_{{\tilde{\alpha }}_{q}}\right)\right)}^{\delta_{q}}+\left(t-1\right)\prod_{q=1}^{n}{\left({\tilde{\eta }}_{{\tilde{\alpha }}_{q}}\right)}^{\delta_{q}}},\frac{t\prod_{q=1}^{n}{\left({\tilde{\xi }}_{{\tilde{\alpha }}_{q}}\right)}^{\delta_{q}}}{\prod_{q=1}^{n}{\left(1+\left(t-1\right)\left(1-{\tilde{\xi }}_{{\tilde{\alpha }}_{q}}\right)\right)}^{\delta_{q}}+\left(t-1\right)\prod_{q=1}^{n}{\left({\tilde{\xi }}_{{\tilde{\alpha }}_{q}}\right)}^{\delta_{q}}},\right) \end{array}$$

**Property** **1.** *Suppose that ${\tilde{\alpha }}_{q}=\left({\tilde{\mu }}_{{\tilde{\alpha }}_{q}},{\tilde{\eta }}_{{\tilde{\alpha }}_{q}},{\tilde{\xi }}_{{\tilde{\alpha }}_{q}}\right)\left(q=1\left(1\right)n\right)$ is a set of PF numbers. If all PF numbers are equal, namely, ${\tilde{\alpha }}_{q}=\tilde{\alpha },\forall q$, then one has*

$$\begin{array}{c} PFGDWA\left({\tilde{\alpha }}_{1},{\tilde{\alpha }}_{2},\cdots ,{\tilde{\alpha }}_{n}\right)=\tilde{\alpha }. \end{array}$$

**Proof.**  The proof of Property 1 is displayed in Appendix C. □

**Property** **2.** *Suppose that ${\tilde{\alpha }}_{q}=\left({\tilde{\mu }}_{{\tilde{\alpha }}_{q}},{\tilde{\eta }}_{{\tilde{\alpha }}_{q}},{\tilde{\xi }}_{{\tilde{\alpha }}_{q}}\right)\left(q=1\left(1\right)n\right)$ and ${\tilde{\tilde{\alpha }}}_{q}=\left({\tilde{\tilde{\mu }}}_{{\tilde{\tilde{\alpha }}}_{q}},{\tilde{\tilde{\eta }}}_{{\tilde{\tilde{\alpha }}}_{q}},{\tilde{\tilde{\xi }}}_{{\tilde{\tilde{\alpha }}}_{q}}\right)$ are two PF numbers such that ${\tilde{\mu }}_{{\tilde{\alpha }}_{q}}\geq {\tilde{\tilde{\eta }}}_{{\tilde{\tilde{\alpha }}}_{q}}$, ${\tilde{\eta }}_{{\tilde{\alpha }}_{q}}\leq {\tilde{\tilde{\eta }}}_{{\tilde{\tilde{\alpha }}}_{q}}$, ${\tilde{\xi }}_{{\tilde{\alpha }}_{q}}\leq {\tilde{\tilde{\xi }}}_{{\tilde{\tilde{\alpha }}}_{q}}$. Then*

$$\begin{array}{c} PFGDWA\left({\tilde{\alpha }}_{1},{\tilde{\alpha }}_{2},\cdots ,{\tilde{\alpha }}_{n}\right)\geq PFGDWA\left({\tilde{\tilde{\alpha }}}_{1},{\tilde{\tilde{\alpha }}}_{2},\cdots ,{\tilde{\tilde{\alpha }}}_{n}\right). \end{array}$$

**Proof.**  The proof of Property 2 is displayed in Appendix D. □

**Property** **3.** *Ponder a set of PF numbers ${\tilde{\alpha }}_{q}=\left({\tilde{\mu }}_{{\tilde{\alpha }}_{q}},{\tilde{\eta }}_{{\tilde{\alpha }}_{q}},{\tilde{\xi }}_{{\tilde{\alpha }}_{q}}\right)\left(q=1\left(1\right)n\right)$ and let ${\tilde{\alpha }}^{-}=\min_{q}{\tilde{\alpha }}_{q}$ and ${\tilde{\alpha }}^{+}=\max_{q}{\tilde{\alpha }}_{q}$. Then,*

$$\begin{array}{c} {\tilde{\alpha }}^{-}\leq PFGDWA\left({\tilde{\alpha }}_{1},{\tilde{\alpha }}_{2},\cdots ,{\tilde{\alpha }}_{n}\right)\leq {\tilde{\alpha }}^{+}. \end{array}$$

**Proof.**  The proof of Property 3 is displayed in Appendix E. □

**Definition** **10.** *Given a collection of PF numbers ${\tilde{\alpha }}_{q}=\left({\tilde{\mu }}_{{\tilde{\alpha }}_{q}},{\tilde{\eta }}_{{\tilde{\alpha }}_{q}},{\tilde{\xi }}_{{\tilde{\alpha }}_{q}}\right)\left(q=1\left(1\right)n\right)$, $\delta ={\left(\delta_{1},\delta_{2},\cdots ,\delta_{n}\right)}^{T}$ is the weight vector of ${\tilde{\alpha }}_{q}\left(q=1\left(1\right)n\right)$, providing that $\delta_{q}>0$ and $\sum_{q=1}^{n}\delta_{q}=1$. Then, the PFGDOWA operator is a mapping from $\Omega^{n}$ to Ω, expressed as*

(18) $$\begin{array}{c} PFGDOWA\left({\tilde{\alpha }}_{1},{\tilde{\alpha }}_{2},\cdots ,{\tilde{\alpha }}_{n}\right)=\overset{n}{\underset{q=1}{\tilde{⊕}}}\left(\delta_{q}{\tilde{\alpha }}_{\varsigma (q)}\right). \end{array}$$

*where $\left(\varsigma (1),\varsigma (2),\cdots ,\varsigma (n)\right)$ signifies the permutation of $\left(1,2,\cdots ,n\right)$ with $\delta_{\varsigma (i-1)}\geq \delta_{\varsigma (i)}$, ∀i=2,3,⋯,n.*

**Theorem** **4.** *The fused value by utilizing the PFGDOWA operator is still a PF number, expressed as*

(19) $$\begin{array}{cc} \; & PFGDOWA\left({\tilde{\alpha }}_{1},{\tilde{\alpha }}_{2},\cdots ,{\tilde{\alpha }}_{n}\right)=\overset{n}{\underset{q=1}{\tilde{⊕}}}\left(\delta_{q}{\tilde{\alpha }}_{\varsigma (q)}\right)\\ \; & \left({\left(1+{\left(\frac{1}{t}\left(\prod_{q=1}^{n}{\left(\Phi_{t}^{s}\left({\tilde{\mu }}_{{\tilde{\alpha }}_{\varsigma (q)}}\right)\right)}^{\delta_{q}}-1\right)\right)}^{-\frac{1}{s}}\right)}^{-1},{\left(1+{\left(\frac{1}{t}\left(\prod_{q=1}^{n}{\left(\Psi_{t}^{s}\left({\tilde{\eta }}_{{\tilde{\alpha }}_{\varsigma (q)}}\right)\right)}^{\delta_{q}}-1\right)\right)}^{\frac{1}{s}}\right)}^{-1},{\left(1+{\left(\frac{1}{t}\left(\prod_{q=1}^{n}{\left(\Psi_{t}^{s}\left({\tilde{\xi }}_{{\tilde{\alpha }}_{\varsigma (q)}}\right)\right)}^{\delta_{q}}-1\right)\right)}^{\frac{1}{s}}\right)}^{-1}\right). \end{array}$$

**Proof.**  Analogous to Theorem 3. □

**Remark** **1.** *(a) The PFGDOWA operator will yield to the PFOWA operator when s=1 and t=1; (b) The PFGDOWA operator will reduce to the PFEOWA operator when s=1 and t=2; (c) The PFGDOWA operator will degenerate into the PFHOWA operator when s=1.*

In view of the aforementioned theorems, the following properties can be attained.

**Property** **4.** *Suppose that ${\tilde{\alpha }}_{q}=\left({\tilde{\mu }}_{{\tilde{\alpha }}_{q}},{\tilde{\eta }}_{{\tilde{\alpha }}_{q}},{\tilde{\xi }}_{{\tilde{\alpha }}_{q}}\right)\left(q=1\left(1\right)n\right)$ and ${\tilde{\tilde{\alpha }}}_{q}=\left({\tilde{\tilde{\mu }}}_{{\tilde{\tilde{\alpha }}}_{q}},{\tilde{\tilde{\eta }}}_{{\tilde{\tilde{\alpha }}}_{q}},{\tilde{\tilde{\xi }}}_{{\tilde{\tilde{\alpha }}}_{q}}\right)$ are two PF numbers. Then, the following properties hold:*

 *1.* *If all PF numbers are equal, namely, ${\tilde{\alpha }}_{q}=\tilde{\alpha },\forall q$, then one has $PFGDOWA\left({\tilde{\alpha }}_{1},{\tilde{\alpha }}_{2},\cdots ,{\tilde{\alpha }}_{n}\right)=\tilde{\alpha }.$*
 *2.* *If ${\tilde{\alpha }}^{-}=\min_{q}{\tilde{\alpha }}_{q}$ and ${\tilde{\alpha }}^{+}=\max_{q}{\tilde{\alpha }}_{q}$, then ${\tilde{\alpha }}^{-}\leq PFGDOWA\left({\tilde{\alpha }}_{1},{\tilde{\alpha }}_{2},\cdots ,{\tilde{\alpha }}_{n}\right)\leq {\tilde{\alpha }}^{+}.$*
 *3.* *If ${\tilde{\alpha }}_{q}\leq {\tilde{\tilde{\alpha }}}_{q},\forall q$, then $PFGDOWA\left({\tilde{\alpha }}_{1},{\tilde{\alpha }}_{2},\cdots ,{\tilde{\alpha }}_{n}\right)\leq PFGDOWA\left({\tilde{\tilde{\alpha }}}_{1},{\tilde{\tilde{\alpha }}}_{2},\cdots ,{\tilde{\tilde{\alpha }}}_{n}\right).$*

### 4.3. Some PF Generalized Dombi Weighted Geometric Operators

In light of the PF generalized Dombi operations defined in Definition 8, we propound the PF generalized Dombi weighted geometric operators and their elegant properties.

**Definition** **11.** *Suppose that ${\tilde{\alpha }}_{q}=\left({\tilde{\mu }}_{{\tilde{\alpha }}_{q}},{\tilde{\eta }}_{{\tilde{\alpha }}_{q}},{\tilde{\xi }}_{{\tilde{\alpha }}_{q}}\right)\left(q=1\left(1\right)n\right)$ is a family of PF numbers and $\delta ={\left(\delta_{1},\delta_{2},\cdots ,\delta_{n}\right)}^{T}$ is the weight vector of ${\tilde{\alpha }}_{q}\left(q=1\left(1\right)n\right)$, providing that $\delta_{q}>0$ and $\sum_{q=1}^{n}\delta_{q}=1$. Then, the PFGDWG operator is a mapping from $\Omega^{n}$ to* Ω, *expressed as*

(20) $$\begin{array}{c} PFGDWG\left({\tilde{\alpha }}_{1},{\tilde{\alpha }}_{2},\cdots ,{\tilde{\alpha }}_{n}\right)=\overset{n}{\underset{q=1}{\tilde{⊗}}}{\left({\tilde{\alpha }}_{q}\right)}^{\delta_{q}}. \end{array}$$

**Theorem** **5.** *The fused value via utilizing PFGDWG operator is still a PF number, portrayed as*

(21) $$\begin{array}{cc} \; & PFGDWG\left({\tilde{\alpha }}_{1},{\tilde{\alpha }}_{2},\cdots ,{\tilde{\alpha }}_{n}\right)=\overset{n}{\underset{q=1}{\tilde{⊕}}}{\left({\tilde{\alpha }}_{q}\right)}^{\delta_{q}}\\ \; & \left({\left(1+{\left(\frac{1}{t}\left(\prod_{q=1}^{n}{\left(\Psi_{t}^{s}\left({\tilde{\mu }}_{{\tilde{\alpha }}_{q}}\right)\right)}^{\delta_{q}}-1\right)\right)}^{\frac{1}{s}}\right)}^{-1},{\left(1+{\left(\frac{1}{t}\left(\prod_{q=1}^{n}{\left(\Phi_{t}^{s}\left({\tilde{\eta }}_{{\tilde{\alpha }}_{q}}\right)\right)}^{\delta_{q}}-1\right)\right)}^{-\frac{1}{s}}\right)}^{-1},{\left(1+{\left(\frac{1}{t}\left(\prod_{q=1}^{n}{\left(\Phi_{t}^{s}\left({\tilde{\xi }}_{{\tilde{\alpha }}_{q}}\right)\right)}^{\delta_{q}}-1\right)\right)}^{-\frac{1}{s}}\right)}^{-1}\right). \end{array}$$

**Proof.**  Analogous to Theorem 3. □

**Remark** **2.** *(a) The PFGDWG operator will yield to the PFWG operator when s=1 and t=1; (b) The PFGDWG operator will reduce to the PFEWG operator when s=1 and t=2; (c) The PFGDWG operator will degenerate into the PFHWG operator when s=1.*

**Definition** **12.** *Given a collection of PF numbers ${\tilde{\alpha }}_{q}=\left({\tilde{\mu }}_{{\tilde{\alpha }}_{q}},{\tilde{\eta }}_{{\tilde{\alpha }}_{q}},{\tilde{\xi }}_{{\tilde{\alpha }}_{q}}\right)\left(q=1\left(1\right)n\right)$, $\delta ={\left(\delta_{1},\delta_{2},\cdots ,\delta_{n}\right)}^{T}$ is the weight vector of ${\tilde{\alpha }}_{q}$, providing that $\delta_{q}>0$ and $\sum_{q=1}^{n}\delta_{q}=1$. Then, the pPFGDOWG operator is a mapping from $\Omega^{n}$ to* Ω*, expressed as*

(22) $$\begin{array}{c} PFGDOWG\left({\tilde{\alpha }}_{1},{\tilde{\alpha }}_{2},\cdots ,{\tilde{\alpha }}_{n}\right)=\overset{n}{\underset{q=1}{\tilde{⊗}}}{\left({\tilde{\alpha }}_{\varsigma (q)}\right)}^{\delta_{q}}. \end{array}$$

*where $\left(\varsigma (1),\varsigma (2),\cdots ,\varsigma (n)\right)$ signifies the permutation of $\left(1,2,\cdots ,n\right)$ with $\delta_{\varsigma (i-1)}\geq \delta_{\varsigma (i)}$, ∀i=2,3,⋯,n.*

**Theorem** **6.** *The fused value by utilizing PFGDOWG operator is still a PF number, expressed as*

(23) $$\begin{array}{cc} \; & PFGDOWG\left({\tilde{\alpha }}_{1},{\tilde{\alpha }}_{2},\cdots ,{\tilde{\alpha }}_{n}\right)=\overset{n}{\underset{q=1}{\tilde{⊕}}}{\left({\tilde{\alpha }}_{\varsigma (q)}\right)}^{\delta_{q}}\\ \; & \left({\left(1+{\left(\frac{1}{t}\left(\prod_{q=1}^{n}{\left(\Psi_{t}^{s}\left({\tilde{\mu }}_{{\tilde{\alpha }}_{\varsigma (q)}}\right)\right)}^{\delta_{q}}-1\right)\right)}^{\frac{1}{s}}\right)}^{-1},{\left(1+{\left(\frac{1}{t}\left(\prod_{q=1}^{n}{\left(\Phi_{t}^{s}\left({\tilde{\eta }}_{{\tilde{\alpha }}_{\varsigma (q)}}\right)\right)}^{\delta_{q}}-1\right)\right)}^{-\frac{1}{s}}\right)}^{-1},{\left(1+{\left(\frac{1}{t}\left(\prod_{q=1}^{n}{\left(\Phi_{t}^{s}\left({\tilde{\xi }}_{{\tilde{\alpha }}_{\varsigma (q)}}\right)\right)}^{\delta_{q}}-1\right)\right)}^{-\frac{1}{s}}\right)}^{-1}\right). \end{array}$$

**Remark** **3.** *(a) The PFGDOWG operator will yield to the PFOWG operator when s=1 and t=1; (b) The PFGDOWG operator will reduce to the PFEOWG operator when s=1 and t=2; (c) The PFGDOWG operator will degenerate into the PFHOWG operator when s=1.*

In view of the aforementioned theorems, the following properties can be acquired.

**Property** **5.** *Ponder a set of PF numbers ${\tilde{\alpha }}_{q}=\left({\tilde{\mu }}_{{\tilde{\alpha }}_{q}},{\tilde{\eta }}_{{\tilde{\alpha }}_{q}},{\tilde{\xi }}_{{\tilde{\alpha }}_{q}}\right)\left(q=1\left(1\right)n\right)$. If all ${\tilde{\alpha }}_{q}=\tilde{\alpha },\forall q$, then $PFGDWG\left({\tilde{\alpha }}_{1},{\tilde{\alpha }}_{2},\cdots ,{\tilde{\alpha }}_{n}\right)=\tilde{\alpha }$ and $PFGDOWG\left({\tilde{\alpha }}_{1},{\tilde{\alpha }}_{2},\cdots ,{\tilde{\alpha }}_{n}\right)=\tilde{\alpha }$ hold.*

**Property** **6.** *Ponder a set of PF numbers ${\tilde{\alpha }}_{q}=\left({\tilde{\mu }}_{{\tilde{\alpha }}_{q}},{\tilde{\eta }}_{{\tilde{\alpha }}_{q}},{\tilde{\xi }}_{{\tilde{\alpha }}_{q}}\right)\left(q=1\left(1\right)n\right)$ and let ${\tilde{\alpha }}^{-}=\min_{q}{\tilde{\alpha }}_{q}$ and ${\tilde{\alpha }}^{+}=\max_{q}{\tilde{\alpha }}_{q}$. Then, ${\tilde{\alpha }}^{-}\leq PFGDWG\left({\tilde{\alpha }}_{1},{\tilde{\alpha }}_{2},\cdots ,{\tilde{\alpha }}_{n}\right)\leq {\tilde{\alpha }}^{+}$ and*

*${\tilde{\alpha }}^{-}\leq PFGDOWG\left({\tilde{\alpha }}_{1},{\tilde{\alpha }}_{2},\cdots ,{\tilde{\alpha }}_{n}\right)\leq {\tilde{\alpha }}^{+}$ hold.*

**Property** **7.** *Let ${\tilde{\alpha }}_{q}=\left({\tilde{\mu }}_{{\tilde{\alpha }}_{q}},{\tilde{\eta }}_{{\tilde{\alpha }}_{q}},{\tilde{\xi }}_{{\tilde{\alpha }}_{q}}\right)\left(q=1\left(1\right)n\right)$ and ${\tilde{\tilde{\alpha }}}_{q}=\left({\tilde{\tilde{\mu }}}_{{\tilde{\tilde{\alpha }}}_{q}},{\tilde{\tilde{\eta }}}_{{\tilde{\tilde{\alpha }}}_{q}},{\tilde{\tilde{\xi }}}_{{\tilde{\tilde{\alpha }}}_{q}}\right)$ be two PF numbers, and ${\tilde{\alpha }}_{q}\leq {\tilde{\tilde{\alpha }}}_{q}$. Then, $PFGDWG\left({\tilde{\alpha }}_{1},{\tilde{\alpha }}_{2},\cdots ,{\tilde{\alpha }}_{n}\right)\leq PFGDWG\left({\tilde{\tilde{\alpha }}}_{1},{\tilde{\tilde{\alpha }}}_{2},\cdots ,{\tilde{\tilde{\alpha }}}_{n}\right)$ and*

*$PFGDOWG\left({\tilde{\alpha }}_{1},{\tilde{\alpha }}_{2},\cdots ,{\tilde{\alpha }}_{n}\right)\leq PFGDOWG\left({\tilde{\tilde{\alpha }}}_{1},{\tilde{\tilde{\alpha }}}_{2},\cdots ,{\tilde{\tilde{\alpha }}}_{n}\right)$ hold.*

## 5. Hybrid PF-PSI-SWARA-CoCoSo Approach

**Figure 1:**
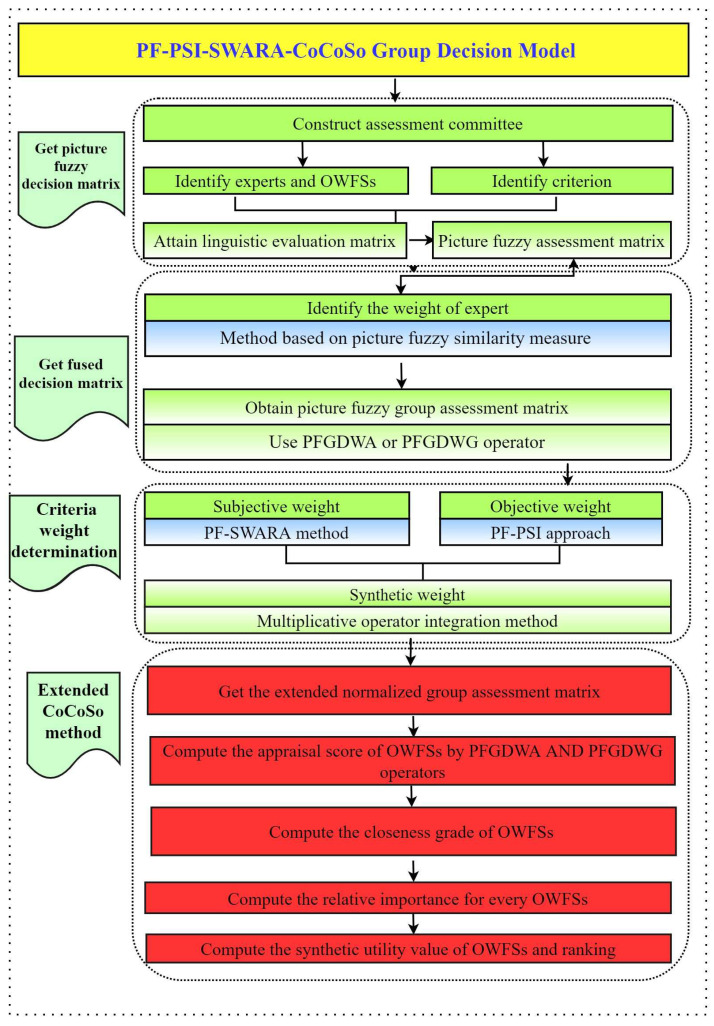
Propounded hybrid PF-PSI-SWARA-CoCoSo approach for selecting OWFSs.

The CoCoSo method is regarded as an efficient approach to acquire the prioritization of the alternatives of uncertain MCDM issues with diverse criteria. The core of the CoCoSo method is to obtain the comprised value of alternatives based on the integration of three aggregation strategies. The objective of this part is to propound a hybrid framework based upon the integration of PSI, SWARA, and the CoCoSo method, called PF-PSI-SWARA-CoCoSo (Figure 1). The detailed decision processes of the designed decision framework are illustrated as follows:

**Step 1:** Establish the linguistic assessment matrix.

In the procedure of constructing the MCGDM methodology within the PF environment, we consider a group of schemes denoted as $Y=\left\{Y_{1},Y_{2},\cdots ,Y_{m}\right\}$ and criteria indicated as $C=\left\{C_{1},C_{2},\cdots ,C_{n}\right\}$. An assessment committee consists of several experts denoted as $E=\left\{E_{1},E_{2},\cdots ,E_{r}\right\}$ and provides their assessment opinion for every alternative $Y_{p}$ with respect to diverse criteria through the linguistic assessment terms. It is supposed that ${\tilde{\Theta }}^{\left(g\right)}={\left({\tilde{h}}_{pq}^{\left(g\right)}\right)}_{m\times n}={\left({\tilde{\mu }}_{pq}^{\left(g\right)},{\tilde{\eta }}_{pq}^{\left(g\right)},{\tilde{\xi }}_{pq}^{\left(g\right)}\right)}_{m\times n}$, p=1(1)m,q=1(1)n is a linguistic assessment matrix provided by experts, in which ${\tilde{h}}_{pq}^{\left(g\right)}$ signifies the assessment standpoint of a schemes $Y_{p}$ over each criteria $C_{q}$ for $g^{th}$ expert.

**Step 2:** Deduce the weight of the experts.

In the process of MCGDM, the importance of an expert is vital for fusing the PF assessments of experts and improving the consistency. Based on the thought that the individual evaluation opinions should be similar to group evaluation to the greatest extent, we utilized the proposed PFGDWA operator and distance measure to improve the similarity-based method for determining the weight of experts. The concrete steps are demonstrated as follows.

**Step 2-1:** Attain the averaging assessment matrix.

Based on the principle that determines the expert weights using the similarity-based method, the expert should possess a greater weight when the similarity degree between expert assessment and group assessment is bigger. Herein, the averaging assessment matrix can be viewed as the group assessment to identify the weight of experts. The averaging assessment matrix ${\tilde{\Theta }}^{N}={\left({\tilde{h}}_{pq}^{N}\right)}_{m\times n}$ can be ascertained by employing the PFGDWA operator, wherein

(24) $$\begin{array}{cc} \; & {\tilde{h}}_{pq}^{N}=PFGDWA\left({\tilde{h}}_{pq}^{\left(1\right)},{\tilde{h}}_{pq}^{\left(2\right)},\cdots ,{\tilde{h}}_{pq}^{\left(r\right)}\right)\\ \; & =\left({\left(1+{\left(\frac{1}{t}\left(\prod_{g=1}^{r}{\left(\Phi_{t}^{s}\left({\tilde{\mu }}_{pq}^{\left(g\right)}\right)\right)}^{\frac{1}{r}}-1\right)\right)}^{-\frac{1}{s}}\right)}^{-1},{\left(1+{\left(\frac{1}{t}\left(\prod_{g=1}^{r}{\left(\Psi_{t}^{s}\left({\tilde{\eta }}_{pq}^{\left(g\right)}\right)\right)}^{\frac{1}{r}}-1\right)\right)}^{\frac{1}{s}}\right)}^{-1},{\left(1+{\left(\frac{1}{t}\left(\prod_{g=1}^{r}{\left(\Psi_{t}^{s}\left({\tilde{\xi }}_{pq}^{\left(g\right)}\right)\right)}^{\frac{1}{r}}-1\right)\right)}^{\frac{1}{s}}\right)}^{-1}\right). \end{array}$$

**Step 2-2:** Compute the similarity degree between the averaging assessment matrix and expert matrices.

The similarity degree $SM\left({\tilde{h}}_{pq}^{\left(g\right)},{\tilde{h}}_{pq}^{N}\right)$ between the average assessment matrix ${\tilde{\Theta }}^{N}={\left({\tilde{h}}_{pq}^{N}\right)}_{m\times n}$ and experts’ assessment matrices ${\tilde{\Theta }}^{\left(g\right)}={\left({\tilde{h}}_{pq}^{\left(g\right)}\right)}_{m\times n}$ is calculated by

(25) $$\begin{array}{c} SM\left({\tilde{h}}_{pq}^{\left(g\right)},{\tilde{h}}_{pq}^{N}\right)=\frac{\sum_{p=1}^{m}\sum_{q=1}^{n}\left[\min\left\{{\tilde{\mu }}_{pq}^{\left(g\right)},{\tilde{\mu }}_{pq}^{N}\right\}+\min\left\{\left(1-{\tilde{\eta }}_{pq}^{\left(g\right)}\right),\left(1-{\tilde{\eta }}_{pq}^{\left(N\right)}\right)\right\}+\min\left\{\left(1-{\tilde{\xi }}_{pq}^{\left(g\right)}\right),\left(1-{\tilde{\xi }}_{pq}^{\left(N\right)}\right)\right\}\right]}{\sum_{p=1}^{m}\sum_{q=1}^{n}\left[\max\left\{{\tilde{\mu }}_{pq}^{\left(g\right)},{\tilde{\mu }}_{pq}^{N}\right\}+\max\left\{\left(1-{\tilde{\eta }}_{pq}^{\left(g\right)}\right),\left(1-{\tilde{\eta }}_{pq}^{\left(N\right)}\right)\right\}+\max\left\{\left(1-{\tilde{\xi }}_{pq}^{\left(g\right)}\right),\left(1-{\tilde{\xi }}_{pq}^{\left(N\right)}\right)\right\}\right]}. \end{array}$$

where $SM\left({\tilde{h}}_{pq}^{\left(g\right)},{\tilde{h}}_{pq}^{N}\right)$ is the propounded PF similarity measure.

**Step 2-3:** Estimate the weight of experts.

The weight of experts can be figured out by the following formulation:

(26) $$\begin{array}{c} \nu_{g}=\frac{SM\left({\tilde{h}}_{pq}^{\left(g\right)},{\tilde{h}}_{pq}^{N}\right)}{\sum_{g=1}^{r}SM\left({\tilde{h}}_{pq}^{\left(g\right)},{\tilde{h}}_{pq}^{N}\right)}, \end{array}$$

where $\nu_{g}$ is the weight of the gth expert, meeting $\nu_{g}>0$ and $\sum_{g=1}^{r}\nu_{g}=1$.

**Step 3:** Fuse the PF assessment matrices of experts.

In order to form the PF group assessment matrix $\tilde{\Theta }={\left({\tilde{h}}_{pq}\right)}_{m\times n}={\left({\tilde{\mu }}_{pq},{\tilde{\eta }}_{pq},{\tilde{\xi }}_{pq}\right)}_{m\times n}$, the experts’ assessment matrices should be amalgamated to a single matrix by utilizing the PFGDWA (or PFGDWG) operator, wherein

(27) $$\begin{array}{cc} \; & {\tilde{h}}_{pq}=\left({\tilde{\mu }}_{pq},{\tilde{\eta }}_{pq},{\tilde{\xi }}_{pq}\right)=PFGDWA\left({\tilde{h}}_{pq}^{\left(1\right)},{\tilde{h}}_{pq}^{\left(2\right)},\cdots ,{\tilde{h}}_{pq}^{\left(r\right)}\right)\\ \; & =\left({\left(1+{\left(\frac{1}{t}\left(\prod_{g=1}^{r}{\left(\Phi_{t}^{s}\left({\tilde{\mu }}_{pq}^{\left(g\right)}\right)\right)}^{\nu_{g}}-1\right)\right)}^{-\frac{1}{s}}\right)}^{-1},{\left(1+{\left(\frac{1}{t}\left(\prod_{g=1}^{r}{\left(\Psi_{t}^{s}\left({\tilde{\eta }}_{pq}^{\left(g\right)}\right)\right)}^{\nu_{g}}-1\right)\right)}^{\frac{1}{s}}\right)}^{-1},{\left(1+{\left(\frac{1}{t}\left(\prod_{g=1}^{r}{\left(\Psi_{t}^{s}\left({\tilde{\xi }}_{pq}^{\left(g\right)}\right)\right)}^{\nu_{g}}-1\right)\right)}^{\frac{1}{s}}\right)}^{-1}\right). \end{array}$$

or

(28) $$\begin{array}{cc} \; & {\tilde{h}}_{pq}=\left({\tilde{\mu }}_{pq},{\tilde{\eta }}_{pq},{\tilde{\xi }}_{pq}\right)=PFGDWG\left({\tilde{h}}_{pq}^{\left(1\right)},{\tilde{h}}_{pq}^{\left(2\right)},\cdots ,{\tilde{h}}_{pq}^{\left(r\right)}\right)\\ \; & =\left({\left(1+{\left(\frac{1}{t}\left(\prod_{g=1}^{r}{\left(\Psi_{t}^{s}\left({\tilde{\mu }}_{pq}^{\left(g\right)}\right)\right)}^{\nu_{g}}-1\right)\right)}^{\frac{1}{s}}\right)}^{-1},{\left(1+{\left(\frac{1}{t}\left(\prod_{g=1}^{r}{\left(\Phi_{t}^{s}\left({\tilde{\eta }}_{pq}^{\left(g\right)}\right)\right)}^{\nu_{g}}-1\right)\right)}^{-\frac{1}{s}}\right)}^{-1},{\left(1+{\left(\frac{1}{t}\left(\prod_{g=1}^{r}{\left(\Phi_{t}^{s}\left({\tilde{\xi }}_{pq}^{\left(g\right)}\right)\right)}^{\nu_{g}}-1\right)\right)}^{-\frac{1}{s}}\right)}^{-1}\right). \end{array}$$

**Step 4:** Derive the weight of assessment criteria by an integrated weight determination model.

**Model-1:** Objective weights identification via PF-PSI method.

The PSI method is an efficient and easy-to-operate approach to identify the importance of criteria in decision analysis [56]. The biggest merit of the PSI model is that the inherent conflict between criteria caused by experts for a decision can be conquered effectively. Thus, the classical PSI method is improved to ascertain the criteria weight under PF scenarios. The concrete procedure of the PF-PSI method is depicted as follows.

**Step 4-1:** Determine the average performance value of all schemes.

Based on the proposed PFGDWA operator, the average performance value $N_{q}$ of all schemes $Y_{p}$ for every criteria $C_{q}$ is calculated by

(29) $$\begin{array}{cc} \; & N_{q}=PFGDWA\left({\tilde{h}}_{1q},{\tilde{h}}_{2q},\cdots ,{\tilde{h}}_{mq}\right)\\ \; & =\left({\left(1+{\left(\frac{1}{t}\left(\prod_{g=1}^{r}{\left(\Phi_{t}^{s}\left({\tilde{\mu }}_{pq}^{\left(g\right)}\right)\right)}^{\frac{1}{m}}-1\right)\right)}^{-\frac{1}{s}}\right)}^{-1},{\left(1+{\left(\frac{1}{t}\left(\prod_{g=1}^{r}{\left(\Psi_{t}^{s}\left({\tilde{\eta }}_{pq}^{\left(g\right)}\right)\right)}^{\frac{1}{m}}-1\right)\right)}^{\frac{1}{s}}\right)}^{-1},{\left(1+{\left(\frac{1}{t}\left(\prod_{g=1}^{r}{\left(\Psi_{t}^{s}\left({\tilde{\xi }}_{pq}^{\left(g\right)}\right)\right)}^{\frac{1}{m}}-1\right)\right)}^{\frac{1}{s}}\right)}^{-1}\right). \end{array}$$

**Step 4-2:** Acquire the variation value of all schemes.

The variation value $\varrho_{q}$ of all alternatives $Y_{p}$ for every criteria $C_{q}$ can be identified by the following formulation:

(30) $$\begin{array}{c} \varrho_{q}=\sum_{p=1}^{m}\left(\tilde{SF}\left({\tilde{h}}_{pq}\right)-\tilde{SF}\left(N_{q}\right)\right),q=1\left(1\right)n. \end{array}$$

**Step 4-3:** Obtain the objective weight of criteria.

Based on the ideal of PSI method, the larger the variation value of $\varrho_{q}$, the larger the weight of criteria $C_{q}$ should be. Hence, the objective weight $\delta_{q}^{s}$ of criteria $C_{q}$ can be figured out by the following formulation:

(31) $$\begin{array}{c} \delta_{q}^{o}=\frac{\varrho_{q}}{\sum_{q=1}^{n}\varrho_{q}},q=1\left(1\right)n, \end{array}$$

where $\delta_{q}^{o}$ is the weight of $q^{th}$ criteria, meeting $\delta_{q}>0$ and $\sum_{q=1}^{n}\delta_{q}=1$.

**Model-2:** Subjective weights identification via PF-SWARA model.

The SWARA method is a robust and advantageous method to identify the criteria weight with the help of the subjective preference of experts for criteria [57]. The merit of the SWARA method is to estimate the opinions of experts on the criteria significance appraisal in the process of determining the weight. This study propounds the PF-SWARA method for computing the criteria weight and gives the concrete steps as follows.

**Step 4-4:** The experts provide their subjective important degree of criteria by linguistic variables and then obtain the aggregation preference of criteria. Further, the crisp values of the aggregation preference for every criteria are computed by the score function.

**Step 4-5:** The criteria are sorted based on the experts’ preference from the most significant to least significant.

**Step 4-6:** Estimate the comparative coefficient $\beta_{q}$ by the following formulation:

(32) $$\beta_{q}=\left\{\begin{array}{cc} 1, & q=1\\ \epsilon_{q}+1, & q>1 \end{array}\right.$$

where $\epsilon_{q}$ signifies the significant grade of the $q^{th}$ criteria.

**Step 4-7:** Compute the weight. The recalculated weight $t_{q}$ is defined by

(33) $$t_{q}=\left\{\begin{array}{cc} 1, & q=1\\ \frac{t_{q-1}}{\beta_{q}}, & q>1 \end{array}\right.$$

**Step 4-8:** Evaluate the normalized criteria weights as

(34) $$\begin{array}{c} \delta_{q}^{s}=\frac{t_{q}}{\sum_{q=1}^{n}t_{q}}. \end{array}$$

**Model-3:** To acquire the integrated weights utilizing PF-PSI-SWARA.

In this article, a synthetic method through synthesizing the PF-PSI model and PF-SWARA method to identify the final integrated weight of criteria is proposed, which can be computed by the following formulation:

(35) $$\begin{array}{c} \delta_{q}=\frac{\sqrt{\left(\delta_{q}^{s}\right)\left(\delta_{q}^{o}\right)}}{\sum_{q=1}^{n}\sqrt{\left(\delta_{q}^{s}\right)\left(\delta_{q}^{o}\right)}}, \end{array}$$

**Step 5:** Establish the normalized group assessment matrix.

The normalized group assessment matrix $\Theta ={\left(h_{pq}\right)}_{m\times n}=\left(\mu_{pq},\eta_{pq},\xi_{pq}\right)$ can be ascertained by

(36) $$\begin{array}{c} h_{pq}=\left\{\begin{array}{cc} \left({\tilde{\mu }}_{pq},{\tilde{\eta }}_{pq},{\tilde{\xi }}_{pq}\right), & \;\mathrm{for}\;j\in C_{b},\\ \left({\tilde{\xi }}_{pq},{\tilde{\eta }}_{pq},{\tilde{\mu }}_{pq}\right), & \;\mathrm{for}\;j\in C_{n}. \end{array}\right., \end{array}$$

where $C_{b}$ and $C_{n}$ denote the benefit and cost criteria, respectively.

**Step 6:** Build up the extended PF group decision matrix.

(37) $$\overset{˘}{h}=\begin{array}{c} \begin{array}{cccc} \;Y_{1} & \;\;\;Y_{2} & \;\;\;\cdots & \;\;\;Y_{n} \end{array}\\ \begin{array}{c} NIS\\ C_{1}\\ \vdots \\ C_{m}\\ PIS \end{array}\left(\begin{array}{cccc} h_{1}^{NIS} & \;h_{2}^{NIS} & \;\cdots & \;h_{n}^{NIS}\\ h_{11} & \;h_{12} & \;\cdots & \;h_{1n}\\ \vdots & \;\vdots & \; & \;\vdots \\ h_{m1} & \;h_{m2} & \;\cdots & \;h_{mn}\\ h_{1}^{PIS} & \;h_{2}^{PIS} & \;\cdots & \;h_{n}^{PIS} \end{array}\right) \end{array},$$

where NIS and PIS denote the negative and positive ideal solutions, respectively, namely, $h_{q}^{NIS}=\left(\min_{1\leq p\leq m}\left\{\mu_{pq}\right\},\min_{1\leq p\leq m}\left\{\eta_{pq}\right\},\max_{1\leq p\leq m}\left\{\xi_{pq}\right\}\right)$, $h_{q}^{PIS}=\left(\max_{1\leq p\leq m}\left\{\mu_{pq}\right\},\max_{1\leq p\leq m}\left\{\eta_{pq}\right\},\min_{1\leq p\leq m}\left\{\xi_{pq}\right\}\right)$.

**Step 7:** Compute the appraisal score of all schemes over each criteria.

By the PFGWA operator and PFGWG operators, the appraisal score of all schemes under every criteria can be determined based on the extended PF group decision matrix.

(38) $$\begin{array}{c} \left\{\begin{array}{cc} \; & {ℵ}_{NIS}^{\left(1\right)}=PFGWA\left(h_{1}^{NIS},h_{2}^{NIS},\cdots ,h_{n}^{NIS}\right)\\ \; & =\left({\left(1+{\left(\frac{1}{t}\left(\prod_{q=1}^{n}{\left(\Phi_{t}^{s}\left({\tilde{\mu }}_{q}^{NIS}\right)\right)}^{\delta_{q}}-1\right)\right)}^{-\frac{1}{s}}\right)}^{-1},{\left(1+{\left(\frac{1}{t}\left(\prod_{q=1}^{n}{\left(\Psi_{t}^{s}\left({\tilde{\eta }}_{q}^{NIS}\right)\right)}^{\delta_{q}}-1\right)\right)}^{\frac{1}{s}}\right)}^{-1},{\left(1+{\left(\frac{1}{t}\left(\prod_{q=1}^{n}{\left(\Psi_{t}^{s}\left({\tilde{\xi }}_{q}^{NIS}\right)\right)}^{\delta_{q}}-1\right)\right)}^{\frac{1}{s}}\right)}^{-1}\right).\\ \; & {ℵ}_{p}^{\left(1\right)}=PFGWA\left(h_{p1},h_{p2},\cdots ,h_{pn}\right)\\ \; & =\left({\left(1+{\left(\frac{1}{t}\left(\prod_{q=1}^{n}{\left(\Phi_{t}^{s}\left({\tilde{\mu }}_{pq}\right)\right)}^{\nu_{g}}-1\right)\right)}^{-\frac{1}{s}}\right)}^{-1},{\left(1+{\left(\frac{1}{t}\left(\prod_{q=1}^{n}{\left(\Psi_{t}^{s}\left({\tilde{\eta }}_{pq}\right)\right)}^{\nu_{g}}-1\right)\right)}^{\frac{1}{s}}\right)}^{-1},{\left(1+{\left(\frac{1}{t}\left(\prod_{q=1}^{n}{\left(\Psi_{t}^{s}\left({\tilde{\xi }}_{pq}\right)\right)}^{\nu_{g}}-1\right)\right)}^{\frac{1}{s}}\right)}^{-1}\right).\\ \; & {ℵ}_{PIS}^{\left(1\right)}=PFGWA\left(h_{1}^{PIS},h_{2}^{PIS},\cdots ,h_{n}^{PIS}\right)\\ \; & =\left({\left(1+{\left(\frac{1}{t}\left(\prod_{q=1}^{n}{\left(\Phi_{t}^{s}\left({\tilde{\mu }}_{q}^{PIS}\right)\right)}^{\delta_{q}}-1\right)\right)}^{-\frac{1}{s}}\right)}^{-1},{\left(1+{\left(\frac{1}{t}\left(\prod_{q=1}^{n}{\left(\Psi_{t}^{s}\left({\tilde{\eta }}_{q}^{PIS}\right)\right)}^{\delta_{q}}-1\right)\right)}^{\frac{1}{s}}\right)}^{-1},{\left(1+{\left(\frac{1}{t}\left(\prod_{q=1}^{n}{\left(\Psi_{t}^{s}\left({\tilde{\xi }}_{q}^{PIS}\right)\right)}^{\delta_{q}}-1\right)\right)}^{\frac{1}{s}}\right)}^{-1}\right). \end{array}\right. \end{array}$$

and

(39) $$\begin{array}{c} \left\{\begin{array}{cc} \; & {ℵ}_{NIS}^{\left(2\right)}=PFGWG\left(h_{1}^{NIS},h_{2}^{NIS},\cdots ,h_{n}^{NIS}\right)\\ \; & =\left({\left(1+{\left(\frac{1}{t}\left(\prod_{g=1}^{r}{\left(\Psi_{t}^{s}\left({\tilde{\mu }}_{pq}^{\left(g\right)}\right)\right)}^{\nu_{g}}-1\right)\right)}^{\frac{1}{s}}\right)}^{-1},{\left(1+{\left(\frac{1}{t}\left(\prod_{g=1}^{r}{\left(\Phi_{t}^{s}\left({\tilde{\eta }}_{pq}^{\left(g\right)}\right)\right)}^{\nu_{g}}-1\right)\right)}^{-\frac{1}{s}}\right)}^{-1},{\left(1+{\left(\frac{1}{t}\left(\prod_{g=1}^{r}{\left(\Phi_{t}^{s}\left({\tilde{\xi }}_{pq}^{\left(g\right)}\right)\right)}^{\nu_{g}}-1\right)\right)}^{-\frac{1}{s}}\right)}^{-1}\right).\\ \; & {ℵ}_{p}^{\left(2\right)}=PFGWG\left(h_{p1},h_{p2},\cdots ,h_{pn}\right)\\ \; & =\left({\left(1+{\left(\frac{1}{t}\left(\prod_{g=1}^{r}{\left(\Psi_{t}^{s}\left({\tilde{\mu }}_{pq}^{\left(g\right)}\right)\right)}^{\nu_{g}}-1\right)\right)}^{\frac{1}{s}}\right)}^{-1},{\left(1+{\left(\frac{1}{t}\left(\prod_{g=1}^{r}{\left(\Phi_{t}^{s}\left({\tilde{\eta }}_{pq}^{\left(g\right)}\right)\right)}^{\nu_{g}}-1\right)\right)}^{-\frac{1}{s}}\right)}^{-1},{\left(1+{\left(\frac{1}{t}\left(\prod_{g=1}^{r}{\left(\Phi_{t}^{s}\left({\tilde{\xi }}_{pq}^{\left(g\right)}\right)\right)}^{\nu_{g}}-1\right)\right)}^{-\frac{1}{s}}\right)}^{-1}\right).\\ \; & {ℵ}_{PIS}^{\left(2\right)}=PFGWG\left(h_{1}^{PIS},h_{2}^{PIS},\cdots ,h_{n}^{PIS}\right)\\ \; & =\left({\left(1+{\left(\frac{1}{t}\left(\prod_{g=1}^{r}{\left(\Psi_{t}^{s}\left({\tilde{\mu }}_{pq}^{\left(g\right)}\right)\right)}^{\nu_{g}}-1\right)\right)}^{\frac{1}{s}}\right)}^{-1},{\left(1+{\left(\frac{1}{t}\left(\prod_{g=1}^{r}{\left(\Phi_{t}^{s}\left({\tilde{\eta }}_{pq}^{\left(g\right)}\right)\right)}^{\nu_{g}}-1\right)\right)}^{-\frac{1}{s}}\right)}^{-1},{\left(1+{\left(\frac{1}{t}\left(\prod_{g=1}^{r}{\left(\Phi_{t}^{s}\left({\tilde{\xi }}_{pq}^{\left(g\right)}\right)\right)}^{\nu_{g}}-1\right)\right)}^{-\frac{1}{s}}\right)}^{-1}\right). \end{array}\right. \end{array}$$

**Step 8:** Compute the closeness grade.

The closeness grades and are calculated by the PF similarity measure, displayed as

(40) $$\begin{array}{cc} \; & \vartheta_{p}^{\left(1\right)}=\frac{SM\left({ℵ}_{p}^{\left(1\right)},{ℵ}_{NIS}^{\left(1\right)}\right)}{SM\left({ℵ}_{p}^{\left(1\right)},{ℵ}_{NIS}^{\left(1\right)}\right)+SM\left({ℵ}_{p}^{\left(1\right)},{ℵ}_{PIS}^{\left(1\right)}\right)}, \end{array}$$

(41) $$\begin{array}{cc} \; & \vartheta_{p}^{\left(2\right)}=\frac{SM\left({ℵ}_{p}^{\left(2\right)},{ℵ}_{NIS}^{\left(2\right)}\right)}{SM\left({ℵ}_{p}^{\left(2\right)},{ℵ}_{NIS}^{\left(2\right)}\right)+SM\left({ℵ}_{p}^{\left(2\right)},{ℵ}_{PIS}^{\left(2\right)}\right)}. \end{array}$$

**Step 9:** Acquire the relative importance for every schemes.

The following three integration strategies are utilized to compute the relative importance for every alternative, which signify the following:

(42) $$\begin{array}{cc} \; & K_{p}^{\left(1\right)}=\frac{\vartheta_{p}^{\left(1\right)}+\vartheta_{p}^{\left(2\right)}}{\sum_{p=1}^{m}\left(\vartheta_{p}^{\left(1\right)}+\vartheta_{p}^{\left(2\right)}\right)}, \end{array}$$

(43) $$\begin{array}{cc} \; & K_{p}^{\left(2\right)}=\frac{\vartheta_{p}^{\left(1\right)}}{\min_{1\leq p\leq m}\left\{\vartheta_{p}^{\left(1\right)}\right\}}+\frac{\vartheta_{p}^{\left(2\right)}}{\min_{1\leq p\leq m}\left\{\vartheta_{p}^{\left(2\right)}\right\}}, \end{array}$$

(44) $$\begin{array}{cc} \; & K_{p}^{\left(3\right)}=\frac{\tau \vartheta_{p}^{\left(1\right)}+\left(1-\tau \right)\vartheta_{p}^{\left(2\right)}}{\tau \max_{1\leq p\leq m}\left\{\vartheta_{p}^{\left(1\right)}\right\}+\left(1-\tau \right)\max_{1\leq p\leq m}\left\{\vartheta_{p}^{\left(2\right)}\right\}}, \end{array}$$

where $K_{p}^{\left(1\right)}$ denotes the arithmetic mean of sum of $\vartheta_{p}^{\left(1\right)}$ and $\vartheta_{p}^{\left(1\right)}$ closeness grade, $K_{p}^{\left(2\right)}$ reflects a sum of the relative closeness grade of $\vartheta_{p}^{\left(1\right)}$ and $\vartheta_{p}^{\left(2\right)}$, and $K_{p}^{\left(3\right)}$ reveals a balance compromise closeness grade of $\vartheta_{p}^{\left(1\right)}$ and $\vartheta_{p}^{\left(2\right)}$. It is noted that the parameter τ(0≤τ≤1) in $K_{p}^{\left(3\right)}$ is a decision strategy coefficient and usually τ=0.5.

**Step 10:** Compute the synthetic utility value $K_{p}\left(p=1\left(1\right)m\right)$ for each alternative and the rank of all schemes.

Based on the three integration values of every alternative, the synthetic utility value of each schemes can be determined by

(45) $$\begin{array}{c} K_{p}={\left(K_{p}^{\left(1\right)}\cdot K_{p}^{\left(2\right)}\cdot K_{p}^{\left(3\right)}\right)}^{\frac{1}{3}}+\frac{1}{3}\left(K_{p}^{\left(1\right)}+K_{p}^{\left(2\right)}+K_{p}^{\left(3\right)}\right). \end{array}$$

Then, the order of schemes can be determined based on the principle that the optimal alternative possesses the larger value of $K_{p}\left(p=1\left(1\right)m\right)$.

**Step 11:** End.

## 6. Case Study

This section develops a numerical case that selects the optimal offshore wind farm site to further demonstrate our proposed PF-PSI-SWARA-CoCoSo approach and validate the effectiveness and feasibility of the propounded methodology.

### 6.1. Description of the Case

In order to cope with the energy crisis situation caused by the gradual depletion of traditional energy and the resulting environmental pollution issues, offshore wind power generation, as a momentous branch of renewable energy, has become a new trend in the development of clean energy. Recently, Chinese power enterprises have planned and selected a suitable OWFS near China’s coastline to vigorously develop clean energy. The planning department of the Chinese power enterprises invited four experts and scholars from the fields of sustainable development, new energy research, and wind power generation to form a decision-making group and determine the optimal offshore wind power generation location based on their evaluation preferences. Firstly, the experts initially selected five alternative sites to be represented as $Y=\left\{Y_{1},Y_{2},\cdots ,Y_{5}\right\}$ through consultation with the relevant department experts. Then, through literature research and analysis from the resources, ten evaluation criteria, denoted as $C=\left\{C_{1},C_{2},\cdots ,C_{10}\right\}$, were determined from the perspectives of environment, technology, economy, and society, and their concrete explanations are shown in Table 1. Afterwards, due to the need to consider the uncertain perception of preference information provided by experts in the evaluation, this paper applies the proposed PF-PSI-SWARA-CoCoSo approach to address the problem of OWFS selection.

### 6.2. Process for Selecting the Optimal OWFS

The hybrid PF-PSI-SWARA-CoCoSo approach is employed to cope with the aforementioned case described in Section 6.1, and the detailed computational processes are shown in the following context.

**Steps 1–3:** To begin with, by means of the linguistic assessment terms for PF numbers displayed in Table 2, the invited four experts and scholars serve their linguistic judgments for the considered OWFSs over the criteria; these assessment for all alternative sites are collected in Table 3, and then the linguistic assessments are transformed into the PF numbers. Afterward, the similarity-based approach is adopted to derive the importance of the experts. By the aid of the PF expert assessment matrices, the averaging assessment matrix is obtained by Equation (24), and then the similarity grade between the averaging assessment matrix and expert matrices are computed by Equation (25). Further, the weight of experts are figured by Equation (26), the results are shown as $\nu_{1}=0.2518,\nu_{2}=0.2480,\nu_{3}=0.2484,$ and $\nu_{4}=0.2519.$ Lastly, the fused assessment matrix is attained by using the PFGDWA operator displayed in Equation (27), the outcome is exhibited in Table 4.

**Step 4:** This step uses two models, including the PF-PSI method and PF-SWARA approach, to determine the objective and subjective weights of criteria, respectively.

Model-1: By means of the Equations (29)–(31), the objective weight of criteria by the PF-PSI method can be computed as $\delta_{q}^{o}=\{0.0352,0.0267,0.0269,0.2924,0.2582,0.0321,0.2588,0.0204,$0.0183,0.0310}.

Model-2: To calculate the subjective weight of criteria, the experts first provide their subjective importance judgment of criteria and then obtain the corresponding aggregated score value. Afterward, based on the Equations (32)–(34), the subjective weight of criteria by PF-SWARA method can be calculated. The mentioned computational outcomes are displayed in Table 5 and Table 6.

Model-3: By means of the Equation (35), the integrated weight can be identified as $\delta_{q}=\left\{0.0688,0.0526,0.0501,0.2259,0.2258,0.0504,.1908,0.0460,0.0401,0.0495\right\}$.

**Step 5:** The normalized group group assessment matrix $\Theta ={\left(h_{pq}\right)}_{m\times n}$ can be attained via Equation (36) and is displayed in Table 7.

**Step 6:** The extended group assessment matrix can be determined by means of Equation (37) and is displayed in Table 8.

**Step 7:** The appraisal scores of all schemes under each criteria are computed with the aid of Equations (38) and (39), and the results are listed as follows:

$$\begin{array}{cc} \; & {ℵ}_{NIS}^{\left(1\right)}=\left(0.9903,0.00008,0.0060\right);{ℵ}_{1}^{\left(1\right)}=\left(0.9915,0.00010,0.0050\right);{ℵ}_{2}^{\left(1\right)}=\left(0.9963,0.00008,0.0019\right);\\ & {ℵ}_{3}^{\left(1\right)}=\left(0.9933,0.00010,0.0036\right);{ℵ}_{4}^{\left(1\right)}=\left(0.9932,0.00009,0.0038\right);{ℵ}_{5}^{\left(1\right)}=\left(0.9926,0.00010,0.0042\right);\\ & {ℵ}_{PIS}^{\left(1\right)}=\left(0.9965,0.00011,0.0018\right);{ℵ}_{NIS}^{\left(2\right)}=\left(0.0052,0.5971,0.9916\right);{ℵ}_{1}^{\left(2\right)}=\left(0.0059,0.6699,0.9899\right);\\ & {ℵ}_{2}^{\left(2\right)}=\left(0.0134,0.6093,0.9736\right);{ℵ}_{3}^{\left(2\right)}=\left(0.0075,0.6561,0.9862\right);{ℵ}_{4}^{\left(2\right)}=\left(0.0074,0.6478,0.9870\right);\\ & {ℵ}_{5}^{\left(2\right)}=\left(0.0068,0.6539,0.9882\right);{ℵ}_{PIS}^{\left(2\right)}=\left(0.0143,0.6927,0.9720\right). \end{array}$$

**Step 8–10:** From the outcomes of the appraisal scores of the schemes, two closeness grades can be determined by Equations (40) and (41). Then, the relative importance for each alternative can be computed via Equations (42)–(44). Further, the synthetic utility value of schemes are figured out with the aid of Equation (45). The computational outcomes of the above-mentioned steps are summarized in Table 9. From it, we can obtain that the optimal offshore wind farm site is $Y_{2}$.

### 6.3. Sensibility Analysis

In practice, experts will make reasonable selections of the parameters and criteria weight coefficients in the propounded PF-PSI-SWARA-CoCoSo method to more reasonably reflect their preferences and attitudes in the process of decision analysis. Therefore, this section conducts dynamic testing for the parameters and weight types involved in the proposed method and analyzes the effect of parameter changes and different types of criteria weight on the ranking of OWFSs. In the following, we execute the sensitivity analysis from two angles, including the parameters analysis in the PFGDWA operator and the CoCoSo method, as well as the change in different types of criteria weight.

**Case** **4.** *We discuss the impact of the parameters s and t in the PFGDWA and PFGDWG operators on the final result. In this case, we set parameter τ=0.5 and combined weight to analyze the affect of parameters s and t=3 on the ranking; the associated outcomes are unfolded in Table 10. With the aid of the results, we can find that it can obtain sorting results when using different combinations of parameters s and t. However, when s=1,t≥2 and s=2,t>2, the prioritization of the alternative sites is $Y_{2}≻Y_{4}≻Y_{5}≻Y_{3}≻Y_{1}$, which implies that the propounded PF-PSI-SWARA-CoCoSo method is relatively stable. In the practical application process, experts need to consider their preferences and select multiple different parameter combinations to determine a more stable ranking result based on the corresponding results.*

**Figure 2:**
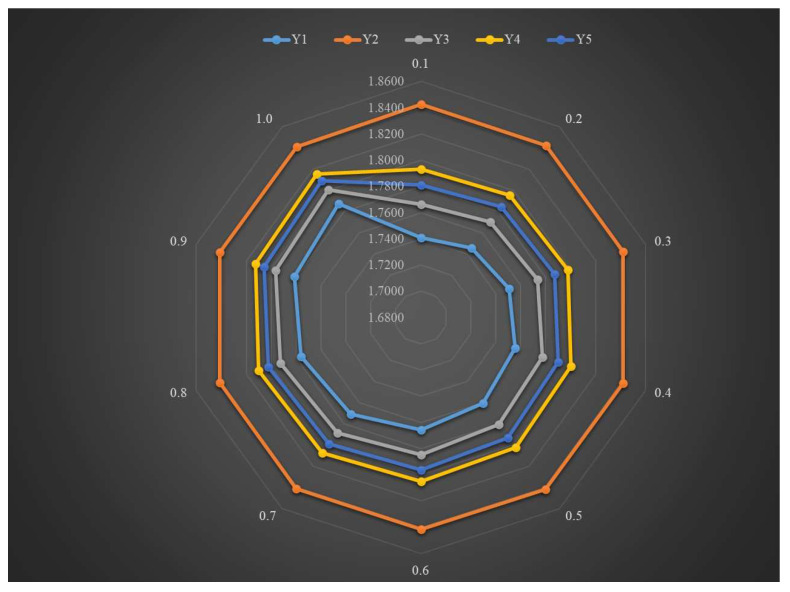
Decision results attained by diverse parameter τ.

**Case** **5.** *We discuss the affect of the parameter τ on the final result. In this case, we set parameter s=2 and t=3 and combined weight to analyze the affect of parameter τ on the ranking; the related outcomes are shown in Table 11 and Figure 2. From the obtained outcomes, we can find that the evaluation values of $Y_{1},Y_{3},Y_{4}$, and $Y_{5}$ gradually increase with the increase in parameter τ, whereas the evaluation values of $Y_{2}$ gradually decrease. However, it is obvious that the best OWFS is always the second site ($Y_{2}$), and the worst option is the first site ($Y_{1}$), when the τ is taken from 0.1 to 1, which implies that the presented PF-PSI-SWARA-CoCoSo method is stable with respect to the parameter τ.*

**Figure 3:**
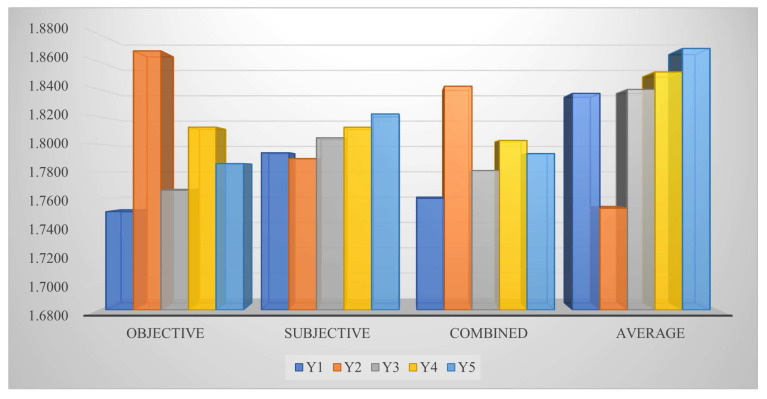
Decision results of diverse types of weight for OWFSs.

**Case** **6.** *We discuss the impact of different types of weights on the final result. The criteria weight is an essential part in the process of decision analysis. Thus, we simulate different types of criteria weights, obtain corresponding ranking results, and then compare the impact of these changes on the decision results. In this case, we set the parameter s=2, t=3, and τ=0.5 and utilize four types of weight in the proposed method to acquire the corresponding assessment values and ranking, respectively, which are exhibited in Table 12 and Figure 3. From the outcomes, we can find that the rankings obtained by subjective and averaging weights are different from the rankings acquired by the objective and combined weights, which indicates that the presented method is sensitive for the criteria weight coefficient.*

### 6.4. Comparison Analysis

**Figure 4:**
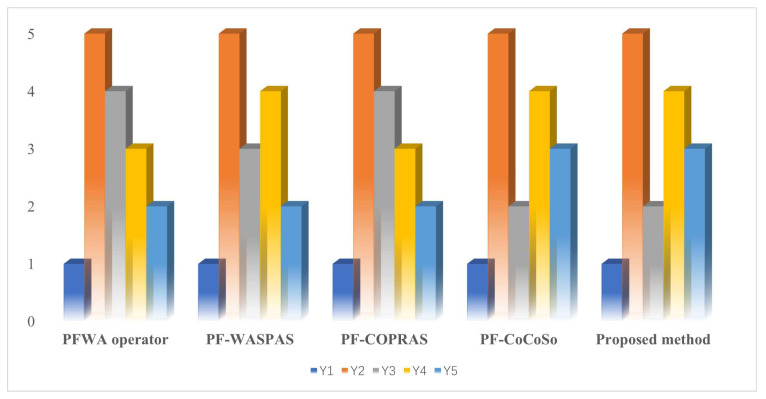
Ranking results of the diverse PF method for OWFSs.

This subsection compares the developed PF-PSI-SWARA-CoCoSo methodology with the extant decision approaches under the PF scenario to further validate the practicability and superiority of our approach. The existing methods include the PFWA-operator-based approach [29], PF-WASPAS method [39], PF-COPRAS [40] approach, and PF-CoSoSo method [41]. In the following, we adopt the mentioned approaches to deal with the case that selects the best OWFS from the alternative option, where the data utilized in the comparison process are the same as this study. The final assessment index of OWFSs by using the mentioned approaches and the corresponding ranking are expounded in Table 13 and Figure 4.

From the comparison outcomes in Table 13 and Figure 4, we can find that although the partial ranks of the site acquired by the extant PF approaches are inconsistent with the presented method, the best option and the worst option obtained by the compared PF methods are the same as those attained utilizing the propounded PF-PSI-SWARA-CoCoSo technique. The results imply that the developed method is effective and feasible for tackling the problem of selecting the OWFS within an uncertain circumstance. In order to further expound the differences and merits of the developed PF-PSI-SWARA-CoCoSo method, a particular comparison between the prior method with our technique is implemented in the following text.

*Comparison with the PF-WASPAS method* [39]. It is built by extending the classical WASPAS method and an assumed criteria weight under the PF environment to cope with the MCDM problem. Although it provides a straightforward way to obtain the rank of schemes, it fails to think over the criteria weight identification and the effect of multiple fusion strategies for the final assessment value. In the proposed PF-PSI-SWARA-CoCoSo technique, the mentioned deficiencies are addressed by different models and an efficient algorithm is provided for experts to unfold decision analysis, which implies that the proposed approach is more universal and feasible than the PF-WASPAS method to tackle real-word decision problems.

*Comparison with the PF-COPRAS method* [40]. It extends the classical COPRAS method to the PF set for building the MCGDM method, but it ignores the determination of expert weight and subjective criteria weight, which may cause information loss in the stage of information fusion. By comparison, the presented PF-PSI-SWARA-CoCoSo offers an integrated decision framework to handle the problem of OWFSs selection, which not only provides the expert and criteria weight determination model but also constructs an enhanced CoCoSo method by the generalized Dombi operators. The proposed approach improves on different perspectives and provides a new group decision framework for tackling decision problems with PF information.

*Comparison with the PF-CoSoSo method* [41]. It is proposed by the combination weight method and CoSoSo method using a novel score function. The PF-CoSoSo method is only employed to settle the MCDM problem, while neglecting the assessment opinions of multiple experts. Furthermore, the PF-CoSoSo method determines the weighted sum and weighted product measures based on the score values of PF evaluations, which may result in missing information in the original PF evaluation. However, the proposed PF-PSI-SWARA-CoCoSo method utilizes the generalized Dombi operators and similarity measure to improve the original weighted sum and weighted product measures, which retains the fuzziness of the initial PF information and makes the final outcomes more reasonable and workable.

Based on the comparison discussion, some characteristic comparisons of the mentioned approach in different decision stages are summarized in Table 14. Further, the strengths of the propounded PF-PSI-SWARA-CoCoSo approach are listed as follows:

(1) The experts weight in the PF-PSI-SWARA-CoCoSo approach is worked out by the similarity-based method, which further ensures the reliability of group assessment opinion.
(2) The proposed method presented the PF-PSI-SWARA method for ascertaining the final criteria weight from the perspective of subjective and objective. In contrast, the PFWA-operator-based method and the PF-WASPAS method suppose that the weight is known. The PF-COPRAS method only considers the objective weight using the CRITIC method. The PF-CoSoSo method ascertains the combined weight by the entropy weight method, and the subjective weight is estimated by experts arbitrarily. Accordingly, the developed PF-PSI-SWARA method for identifying the criteria weight further enhances the reasonability of final ranking outcomes.
(3) The proposed enhanced PF-CoSoSo method using the PF generalized Dombi operator and similarity measure determines the prioritization of schemes by means of three integration strategies, which strengthens the rationality and dependability of the ultimate ranking.

## 7. Conclusions

The problem of selecting a suitable OWFS is regarded as a typical MCDM problem and has become an essential foundation for the development of clean energy for offshore wind power. Therefore, this study propounds a hybrid PF-PSI-SWARA-CoCoSo decision framework for the selection of OWFS. Concretely, we first propose a new similarity measure for PF sets. Next, the novel operations on PF numbers based upon the generalized Dombi norms are defined. Further, a series of novel aggregation operators like the PFGDWA, PFGDWG, PFGDOWA, and PFGDOWG operators is propounded. Furthermore, a hybrid PF-PSI-SWARA-CoCoSo method is constructed based on the developed similarity measure and PF generalized Dombi operators, wherein the criteria weight is ascertained by the presented PF-PSI-SWARA method and the prioritization of schemes is obtained by an enhanced CoCoSo method. A case that aims to choose the best OWFS is implemented to exhibit the feasibility and applicability of the developed decision framework. We also conduct the experiment analysis by including parameter analysis and a comparison study to validate the stability, effectiveness, and tremendous superiority of the advanced hybrid approach. The results show that the presented methodology possess more merits than the extant decision methods under PF context to cope with uncertain decision problems. The determination methods of expert weight and criteria weight are presented by novel theories, which further enhances reasonability and scientificity. The enhanced CoCoSo method under the PF setting also strengths the flexibility of decision. The developed method provides a useful tool for stakeholders in the offshore wind industry to make informed decisions and promote sustainable development.

Nevertheless, several shortcomings also exist in the method when expounding the ranking outcomes. First, the criteria in this study need to be further explored tp build a more comprehensive and systematic assessment index system. Then, the decision committee in practical problems may consist of a large group of experts from various fields. In addition, the interactive impact among criteria also needs to be take into account in the process of actual decision analysis.

Future research will focus on the following four aspects: (1) to establishing a comprehensive evaluation index system for the selection of OWFSs from the perspective of sustainable development and low carbon emissions; (2) to take into account the establishment and application of a large-scale group decision framework in an intelligent linguistic environment [58,59]; (3) to propose new integrated operators based on the proposed operations that consider the interactive impact of the criteria; and, lastly, (4) some new decision models such as double-normalization-based multiple aggregation [60], gained lost dominance score [61], mixed aggregation by comprehensive normalization technique [62], and weighted sum product [63] can be extended to PF environments to enrich their applications in decision methods.

## Figures and Tables

**Table 1:** Criteria for the offshore wind farm site selection.

Dimension	Criteria	Characteristic	Type	Reference
Resource	Wind speed ($C_{1}$)	Quantitative	Positive	[2,3,5,6,7,8,10]
	Wind density ($C_{2}$)	Quantitative	Positive	[2,3,5,6,7,8,10]
	Wind hours ($C_{3}$)	Quantitative	Positive	[3,4,5,6,7,8,10]
Economic	Investment ($C_{4}$)	Quantitative	Negative	[3,4,5,6,7,10,11]
	Payback period ($C_{5}$)	Quantitative	Negative	[3,4,5,6,8,10,11]
Environment	Low carbon emissions ($C_{6}$)	Qualitative	Positive	[3,4,6,8,10,11]
	Noise impact ($C_{7}$)	Qualitative	Negative	[3,4,8,10]
Technical & Social	Traffic condition ($C_{8}$)	Qualitative	Positive	[7,8,10,11]
	Policy support ($C_{9}$)	Qualitative	Positive	[8,10,11]
	Public acceptnce ($C_{10}$)	Qualitative	Positive	[10,11]

**Table 2:** Linguistic assessment terms for the offshore wind farm site selection.

Linguistic Assessment Terms	PF Numbers
Very good (VG)	(0.90, 0.05, 0.05)
Good (G)	(0.75, 0.05, 0.10
Moderately good (MG)	(0.60, 0.05, 0.30)
Fair (F)	(0.50, 0.10, 0.40)
Moderately bad (MB)	(0.30, 0.05, 0.60)
Bad (B)	(0.20, 0.05, 0.70)
Very bad (VB)	(0.10, 0.05, 0.80)

**Table 3:** Individual linguistic matrices given by experts.

Criteria	$Y_{1}$	$Y_{2}$	$Y_{3}$	$Y_{4}$	$Y_{5}$
$C_{1}$	(G, F, MG, MG)	(VG, VG, VG, G)	(G, G, G, G)	(MG, F, F, F)	(F, G, F, MG)
$C_{2}$	(F, F, F, G)	(G, G, G, VG)	(VG, G, F, G)	(G, MG, G, F)	(MG, G, F, G)
$C_{3}$	(G, G, G, G)	(VG, F, VG, VG)	(G, G, F, MG)	(F, F, G, F)	(MG, MG, MG, G)
$C_{4}$	(B, F, MB, F)	(MB, F, VB, VB)	(F, B, B, F)	(B, VB, B, B)	(VB, B, F, F)
$C_{5}$	(B, MB, F, B)	(B, F, VB, B)	(F, B, B, F)	(B, B, F, F)	(VB, F, MB, MB)
$C_{6}$	(F, F, F, F)	(VG, VG, VG, G)	(G, G, G, G)	(G, G, MG, MG)	(VG, F, MG, F)
$C_{7}$	(F, F, B, F)	(B, B, VB, B)	(VB, VB, F, F)	(F, B, F, MB)	(B, F, B, F)
$C_{8}$	(G, F, F, G)	(G, VG, G, G)	(VG, F, VG, F)	(G, G, G, VG)	(F, G, MG, MG)
$C_{9}$	(F, MG, G, G)	(VG, VG, VG, VG)	(G, G, VG, F)	(VG, F, G, G)	(G, G, F, F)
$C_{10}$	(F, G, G, F)	(G, VG, G, VG)	(F, G, MG, MG)	(G, MG, MG, F)	(G, F, MG, G)

**Table 4:** The fused assessment matrix obtained by PFGDWA operator.

Criteria	$Y_{1}$	$Y_{2}$	$Y_{3}$	$Y_{4}$	$Y_{5}$
$C_{1}$	(0.8935, 0.0120, 0.0615)	(0.9726, 0.0100, 0.0121)	(0.9397, 0.0100, 0.0209)	(0.8520, 0.0174, 0.1030)	(0.8831, 0.0145, 0.0686)
$C_{2}$	(0.8727, 0.0174, 0.0755)	(0.9536, 0.0100, 0.0174)	(0.9400, 0.0120, 0.0269)	(0.9087, 0.0121, 0.0448)	(0.9088, 0.0120, 0.0448)
$C_{3}$	(0.9397, 0.0100, 0.0209)	(0.9644, 0.0120, 0.0187)	(0.9088, 0.0120, 0.0448)	(0.8722, 0.0175, 0.0760)	(0.9027, 0.0100, 0.0554)
$C_{4}$	(0.7481, 0.0145, 0.1770)	(0.5831, 0.0120, 0.2783)	(0.7231, 0.0145, 0.1929 )	(0.5151, 0.0100, 0.3392)	(0.6794, 0.0145, 0.2156 )
$C_{5}$	(0.6769, 0.0120, 0.2277)	(0.5996, 0.0120, 0.2734)	(0.7231, 0.0145, 0.1929)	(0.7222, 0.0145, 0.1935)	(0.6617, 0.0120, 0.2321)
$C_{6}$	(0.8386, 0.0209, 0.1137)	(0.9726, 0.0100, 0.0121)	(0.9290, 0.0100, 0.0292)	(0.9168, 0.0100, 0.0403)	(0.9090, 0.0145, 0.0572)
$C_{7}$	(0.7864, 0.0175, 0.1490)	(0.5150, 0.0100, 0.3392)	(0.6341, 0.0145, 0.2390)	(0.7485, 0.0145, 0.1768)	(0.7220, 0.0145, 0.1936)
$C_{8}$	(0.9004, 0.0145, 0.0495)	(0.9534, 0.0100, 0.0175)	(0.9397, 0.0145, 0.0348)	(0.9536, 0.0100, 0.0174)	(0.8931, 0.0121, 0.0619)
$C_{9}$	(0.9087, 0.0121, 0.0448)	(0.9791, 0.0100, 0.0100)	(0.9395, 0.0121, 0.0271)	(0.9400, 0.0120, 0.0269)	(0.9000, 0.0145, 0.0498)
$C_{10}$	(0.8996, 0.0145, 0.0501)	(0.9643, 0.0100, 0.0145)	(0.8931, 0.0121, 0.0619)	(0.8834, 0.0145, 0.0682)	(0.9090, 0.0120, 0.0446)

**Table 5:** Importance evaluation for each criteria by experts.

Criteria	$E_{1}$	$E_{2}$	$E_{3}$	$E_{4}$	Fused PF Numbers	Score Values
$C_{1}$	G	G	VG	F	(0.7636, 0.0595, 0.1189)	1.6968
$C_{2}$	G	G	F	MG	(0.6656, 0.0595, 0.1861)	1.3979
$C_{3}$	G	MG	F	MG	(0.6239, 0.0595, 0.2449)	1.2844
$C_{4}$	G	G	VG	VG	(0.8419, 0.0500, 0.0707)	1.9827
$C_{5}$	VG	VG	G	VG	(0.8743, 0.0500, 0.0595)	2.1141
$C_{6}$	MG	MG	F	F	(0.5528, 0.0707, 0.3464)	1.1118
$C_{7}$	VG	MG	G	G	(0.7764, 0.0500, 0.1107)	1.7414
$C_{8}$	MG	G	G	F	(0.6656, 0.0595, 0.1861)	1.3979
$C_{9}$	MG	F	G	F	(0.6024, 0.0707, 0.2632)	1.2296
$C_{10}$	F	MG	MG	F	(0.5528, 0.0707, 0.3464)	1.1118

**Table 6:** Calculation results of subjective weight by PF-SWARA method.

Criteria	Crisp Grade	Comparison Importance	Coefficient	Recalculated Weight	Subjective Weight
$C_{5}$	2.1141	-	1.0000	1.0000	0.1654
$C_{4}$	1.9827	0.1314	1.1314	0.8838	0.1462
$C_{7}$	1.7414	0.2413	1.2413	0.7120	0.1178
$C_{1}$	1.6968	0.0446	1.0446	0.6816	0.1128
$C_{8}$	1.3979	0.2989	1.2989	0.5248	0.0868
$C_{2}$	1.3979	0.0000	1.0000	0.5248	0.0868
$C_{3}$	1.2844	0.1135	1.1135	0.4713	0.0780
$C_{9}$	1.2296	0.0549	1.0549	0.4468	0.0739
$C_{6}$	1.1118	0.1177	1.1177	0.3997	0.0661
$C_{10}$	1.1118	0.0000	1.0000	0.3997	0.0661

**Table 7:** The normalized group assessment matrix $\Theta ={\left(h_{pq}\right)}_{m\times n}$.

Criteria	$Y_{1}$	$Y_{2}$	$Y_{3}$	$Y_{4}$	$Y_{5}$
$C_{1}$	(0.8935, 0.0120, 0.0615)	(0.9726, 0.0100, 0.0121)	(0.9397, 0.0100, 0.0209)	(0.8520, 0.0174, 0.1030)	(0.8831, 0.0145, 0.0686)
$C_{2}$	(0.8727, 0.0174, 0.0755)	(0.9536, 0.0100, 0.0174)	(0.9400, 0.0120, 0.0269)	(0.9087, 0.0121, 0.0448)	(0.9088, 0.0120, 0.0448)
$C_{3}$	(0.9397, 0.0100, 0.0209)	(0.9644, 0.0120, 0.0187)	(0.9088, 0.0120, 0.0448)	(0.8722, 0.0175, 0.0760)	(0.9027, 0.0100, 0.0554)
$C_{4}$	(0.1770, 0.0145, 0.7481)	(0.2783, 0.0120, 0.5831)	(0.1929, 0.0145, 0.7231)	(0.3392, 0.0100, 0.5151)	(0.2156, 0.0145, 0.6794)
$C_{5}$	(0.2277, 0.0120, 0.6769)	(0.2734, 0.0120, 0.5996)	(0.1929, 0.0145, 0.7231)	(0.1935, 0.0145, 0.7222)	(0.2321, 0.0120, 0.6617)
$C_{6}$	(0.8386, 0.0209, 0.1137)	(0.9726, 0.0100, 0.0121)	(0.9290, 0.0100, 0.0292)	(0.9168, 0.0100, 0.0403)	(0.9090, 0.0145, 0.0572)
$C_{7}$	(0.1490, 0.0175, 0.7864)	(0.3392, 0.0100, 0.5150)	(0.2390, 0.0145, 0.6341)	(0.1768, 0.0145, 0.7485)	(0.1936, 0.0145, 0.7220)
$C_{8}$	(0.9004, 0.0145, 0.0495)	(0.9534, 0.0100, 0.0175)	(0.9397, 0.0145, 0.0348)	(0.9536, 0.0100, 0.0174)	(0.8931, 0.0121, 0.0619)
$C_{9}$	(0.9087, 0.0121, 0.0448)	(0.9791, 0.0100, 0.0100)	(0.9395, 0.0121, 0.0271)	(0.9400, 0.0120, 0.0269)	(0.9000, 0.0145, 0.0498)
$C_{10}$	(0.8996, 0.0145, 0.0501)	(0.9643, 0.0100, 0.0145)	(0.8931, 0.0121, 0.0619)	(0.8834, 0.0145, 0.0682)	(0.9090, 0.0120, 0.0446)

**Table 8:** The extended group assessment matrix.

Criteria	NIS	$Y_{1}$	$Y_{2}$	$Y_{3}$	$Y_{4}$	$Y_{5}$	PIS
$C_{1}$	(0.8520, 0.0100, 0.1030)	(0.8935, 0.0120, 0.0615)	(0.9726, 0.0100, 0.0121)	(0.9397, 0.0100, 0.0209)	(0.8520, 0.0174, 0.1030)	(0.8831, 0.0145, 0.0686)	(0.9726, 0.0174, 0.0121)
$C_{2}$	(0.8727, 0.0100, 0.0755)	(0.8727, 0.0174, 0.0755)	(0.9536, 0.0100, 0.0174)	(0.9400, 0.0120, 0.0269)	(0.9087, 0.0121, 0.0448)	(0.9088, 0.0120, 0.0448)	(0.9536, 0.0174, 0.0174)
$C_{3}$	(0.8722, 0.0100, 0.0760)	(0.9397, 0.0100, 0.0209)	(0.9644, 0.0120, 0.0187)	(0.9088, 0.0120, 0.0448)	(0.8722, 0.0175, 0.0760)	(0.9027, 0.0100, 0.0554)	(0.9644, 0.0175, 0.0187)
$C_{4}$	(0.1770, 0.0100, 0.7481)	(0.1770, 0.0145, 0.7481)	(0.2783, 0.0120, 0.5831)	(0.1929, 0.0145, 0.7231)	(0.3392, 0.0100, 0.5151)	(0.2156, 0.0145, 0.6794)	(0.3392, 0.0145, 0.5151)
$C_{5}$	(0.1929, 0.0120, 0.7231)	(0.2277, 0.0120, 0.6769)	(0.2734, 0.0120, 0.5996)	(0.1929, 0.0145, 0.7231)	(0.1935, 0.0145, 0.7222)	(0.2321, 0.0120, 0.6617)	(0.2734, 0.0145 0.5996)
$C_{6}$	(0.8386, 0.0100, 0.1137)	(0.8386, 0.0209, 0.1137)	(0.9726, 0.0100, 0.0121)	(0.9290, 0.0100, 0.0292)	(0.9168, 0.0100, 0.0403)	(0.9090, 0.0145, 0.0572)	(0.9726, 0.0209, 0.0121)
$C_{7}$	(0.1490, 0.0100, 0.7864)	(0.1490, 0.0175, 0.7864)	(0.3392, 0.0100, 0.5150)	(0.2390, 0.0145, 0.6341)	(0.1768, 0.0145, 0.7485)	(0.1936, 0.0145, 0.7220)	(0.3392, 0.0175, 0.5150)
$C_{8}$	(0.8931, 0.0100, 0.0619)	(0.9004, 0.0145, 0.0495)	(0.9534, 0.0100, 0.0175)	(0.9397, 0.0145, 0.0348)	(0.9536, 0.0100, 0.0174)	(0.8931, 0.0121, 0.0619)	(0.9536, 0.0145, 0.0174)
$C_{9}$	(0.9000, 0.0100, 0.0498)	(0.9087, 0.0121, 0.0448)	(0.9791, 0.0100, 0.0100)	(0.9395, 0.0121, 0.0271)	(0.9400, 0.0120, 0.0269)	(0.9000, 0.0145, 0.0498)	(0.9791, 0.0145, 0.0100)
$C_{10}$	(0.8834, 0.0100, 0.0682)	(0.8996, 0.0145, 0.0501)	(0.9643, 0.0100, 0.0145)	(0.8931, 0.0121, 0.0619)	(0.8834, 0.0145, 0.0682)	(0.9090, 0.0120, 0.0446)	(0.9643, 0.0145, 0.0145)

**Table 9:** The computational outcomes obtained by CoCoSo method.

	$\vartheta_{p}^{\left(1\right)}$	$\vartheta_{p}^{\left(2\right)}$	$K_{p}^{\left(1\right)}$	$K_{p}^{\left(2\right)}$	$K_{p}^{\left(3\right)}$	$K_{p}$	Ranking
$Y_{1}$	0.5005	0.4858	0.1956	1.9788	0.9548	1.7607	5
$Y_{2}$	0.4992	0.5326	0.2046	2.0701	0.9987	1.8419	1
$Y_{3}$	0.5000	0.4977	0.1979	2.0016	0.9657	1.7809	4
$Y_{4}$	0.5000	0.5098	0.2003	2.0260	0.9775	1.8027	2
$Y_{5}$	0.5002	0.5043	0.1992	2.0154	0.9724	1.7932	3

**Table 10:** The impact of σ for the ultimate decision results.

*s*	*t*	Ranking Values					Sorting
		$K_{1}$	$K_{2}$	$K_{3}$	$K_{4}$	$K_{5}$	
	t=1	1.9615	1.6959	1.8573	1.8803	1.9178	$Y_{1}≻Y_{5}≻Y_{4}≻Y_{3}≻Y_{2}$
	t=2	1.7244	1.8837	1.7594	1.7917	1.7718	$Y_{2}≻Y_{4}≻Y_{5}≻Y_{3}≻Y_{1}$
s=1	t=3	1.6973	1.9244	1.7390	1.7943	1.7577	$Y_{2}≻Y_{4}≻Y_{5}≻Y_{3}≻Y_{1}$
	t=4	1.6953	1.9299	1.7362	1.7984	1.7569	$Y_{2}≻Y_{4}≻Y_{5}≻Y_{3}≻Y_{1}$
	t=5	1.6951	1.9314	1.7354	1.8002	1.7568	$Y_{2}≻Y_{4}≻Y_{5}≻Y_{3}≻Y_{1}$
	t=6	1.6951	1.9319	1.7351	1.8011	1.7568	$Y_{2}≻Y_{4}≻Y_{5}≻Y_{3}≻Y_{1}$
	t=1	1.9615	1.6959	1.8573	1.8803	1.9178	$Y_{1}≻Y_{5}≻Y_{4}≻Y_{3}≻Y_{2}$
	t=2	1.8734	1.7509	1.8414	1.8591	1.8697	$Y_{1}≻Y_{5}≻Y_{4}≻Y_{3}≻Y_{2}$
s=2	t=3	1.7607	1.8419	1.7809	1.8027	1.7932	$Y_{2}≻Y_{4}≻Y_{5}≻Y_{3}≻Y_{1}$
	t=4	1.7244	1.8837	1.7594	1.7917	1.7718	$Y_{2}≻Y_{4}≻Y_{5}≻Y_{3}≻Y_{1}$
	t=5	1.7100	1.9030	1.7497	1.7902	1.7640	$Y_{2}≻Y_{4}≻Y_{5}≻Y_{3}≻Y_{1}$
	t=6	1.7035	1.9129	1.7448	1.7909	1.7607	$Y_{2}≻Y_{4}≻Y_{5}≻Y_{3}≻Y_{1}$

**Table 11:** The impact of τ for the ultimate decision results.

τ	Ranking Values					Sorting
	$K_{1}$	$K_{2}$	$K_{3}$	$K_{4}$	$K_{5}$	
0.1	1.7407	1.8425	1.7662	1.7931	1.7812	$Y_{2}≻Y_{4}≻Y_{5}≻Y_{3}≻Y_{1}$
0.2	1.7456	1.8423	1.7698	1.7955	1.7842	$Y_{2}≻Y_{4}≻Y_{5}≻Y_{3}≻Y_{1}$
0.3	1.7506	1.8422	1.7735	1.7979	1.7871	$Y_{2}≻Y_{4}≻Y_{5}≻Y_{3}≻Y_{1}$
0.4	1.7556	1.8420	1.7772	1.8003	1.7901	$Y_{2}≻Y_{4}≻Y_{5}≻Y_{3}≻Y_{1}$
0.5	1.7607	1.8419	1.7809	1.8027	1.7932	$Y_{2}≻Y_{4}≻Y_{5}≻Y_{3}≻Y_{1}$
0.6	1.7659	1.8417	1.7847	1.8051	1.7963	$Y_{2}≻Y_{4}≻Y_{5}≻Y_{3}≻Y_{1}$
0.7	1.7711	1.8416	1.7885	1.8076	1.7994	$Y_{2}≻Y_{4}≻Y_{5}≻Y_{3}≻Y_{1}$
0.8	1.7763	1.8414	1.7924	1.8101	1.8025	$Y_{2}≻Y_{4}≻Y_{5}≻Y_{3}≻Y_{1}$
0.9	1.7816	1.8413	1.7963	1.8127	1.8057	$Y_{2}≻Y_{4}≻Y_{5}≻Y_{3}≻Y_{1}$
1.0	1.7870	1.8411	1.8003	1.8152	1.8089	$Y_{2}≻Y_{4}≻Y_{5}≻Y_{3}≻Y_{1}$

**Table 12:** The impact of different weight types on the ultimate decision results.

Weight Type	Ranking Values					Sorting
	$K_{1}$	$K_{2}$	$K_{3}$	$K_{4}$	$K_{5}$	
Objective weight	1.7511	1.8674	1.7666	1.8123	1.7859	$Y_{2}≻Y_{4}≻Y_{5}≻Y_{3}≻Y_{1}$
Subjective weight	1.7938	1.7896	1.8046	1.8122	1.8218	$Y_{5}≻Y_{4}≻Y_{3}≻Y_{1}≻Y_{2}$
Combined wight	1.7607	1.8419	1.7809	1.8027	1.7932	$Y_{2}≻Y_{4}≻Y_{5}≻Y_{3}≻Y_{1}$
Averaging wight	1.8367	1.7537	1.8396	1.8522	1.8692	$Y_{5}≻Y_{4}≻Y_{3}≻Y_{1}≻Y_{2}$

**Table 13:** Decision results by utilizing different PF decision methodologies.

Approaches	Ranking Values					Sorting
	$K_{1}$	$K_{2}$	$K_{3}$	$K_{4}$	$K_{5}$	
PFWA-operator-based method in [29]	0.3078	0.6008	0.4202	0.3701	0.3341	$Y_{2}≻Y_{3}≻Y_{4}≻Y_{5}≻Y_{1}$
PF-WASPAS method in [39]	0.1470	0.3681	0.2102	0.2217	0.1993	$Y_{2}≻Y_{4}≻Y_{3}≻Y_{5}≻Y_{1}$
PF-COPRAS method in [40]	0.0136	1.0000	0.4294	0.1195	0.0597	$Y_{2}≻Y_{3}≻Y_{4}≻Y_{5}≻Y_{1}$
PF-CoSoSo method in [41]	1.7449	1.8935	1.7887	1.7970	1.7766	$Y_{2}≻Y_{4}≻Y_{5}≻Y_{3}≻Y_{1}$
Propounded method	1.7607	1.8419	1.7809	1.8027	1.7932	$Y_{2}≻Y_{4}≻Y_{5}≻Y_{3}≻Y_{1}$

**Table 14:** Characteristic comparison between the propounded and other PF methods.

Methods	Calculation of Experts Weight	Flexibility of the Fusion Procedure	Criteria Weight	Ranking Algorithm	Consider Multiple Fusion Strategies	MCGDM
PFWA-based method in [29]	NO	NO	Assume	Fusion	NO	NO
PF-WASPAS method in [39]	NO	NO	Assume	WASPAS	NO	NO
PF-COPRAS method in [40]	Assume	NO	CRITIC	COPRAS	NO	YES
PF-CoSoSo method in [41]	NO	NO	CRITIC	CoSoSo	YES	NO
Propounded method	Computing	YES	PSI-SWARA	Enhanced CoSoSo	YES	YES

## Data Availability

The data presented in this study are available in the article.

## Abbreviations

The following abbreviations are used in this manuscript:

**Full Name**	**Abbreviation**
Offshore Wind Farm Site	OWFS
Multicriteria Decision Making	MCDM
Picture Fuzzy	PF
Combined Compromise Solution	CoCoSo
Preference Selection Index	PSI
Stepwise Weights Assessment Ratio Analysis	SWARA
Multicriteria Group Decision Making	MCGDM
Level-Based Weight Assessment	LBWA
Multiattributive Border Approximation area Comparison	MABAC
CRiterion Importance Through Intercriteria Correlation	CRITIC
Technique for Order Preference by Similarity to an Ideal Solution	TOPSIS
Fuzzy Set	FS
Weighted Aggregated Sum Product Assessment	WASPAS
COmplex PRoportional ASsessment	COPRAS
Measurement Alternatives and Ranking according to Compromise Solution	MARCOS
Multiobjective Optimization Based on the Ratio Analysis with the Full Multiplicative Form	MULTIMOORA
Failure Modes and Effects Analysis	FMEA
Picture Fuzzy Generalized Dombi Weighted Averaging	PFGDWA
Picture Fuzzy Generalized Dombi Ordered Weighted Averaging	PFGDOWA
Picture Fuzzy Generalized Dombi Weighted Geometric	PFGDWG
Picture Fuzzy Generalized Dombi Ordered Weighted Geometric	PFGDOWG
Picture Fuzzy Weighted Averaging	PFWA
Picture Fuzzy Einstein Weighted Averaging	PFEWA
Picture Fuzzy Hamacher Weighted Averaging	PFHWA

## Notions

**Mathematical Symbols**	**Explain**
$\tilde{A}$	PF set,
X	the universe of discourse,
${\tilde{\mu }}_{\tilde{A}}\left(\chi \right)$	positive membership function,
${\tilde{\eta }}_{\tilde{A}}\left(\chi \right)$	neutral membership function,
$\xi_{\tilde{A}}\left(\chi \right)$	negative membership function,
${\tilde{\pi }}_{\tilde{A}}\left(\chi \right)$	refusal degree of the PF set $\tilde{A}$,
$\tilde{\alpha }=\left({\tilde{\mu }}_{\tilde{\alpha }},{\tilde{\eta }}_{\tilde{\alpha }},{\tilde{\xi }}_{\tilde{\alpha }}\right)$	PF number,
$SF\left(\tilde{\alpha }\right)$	score function,
$AF\left(\tilde{\alpha }\right)$	accuracy function,
$\tilde{SF}\left(\tilde{\alpha }\right)$	score function,
$SM\left(\tilde{A},\tilde{B}\right)$	similarity degree between PF set $\tilde{A}$ and $\tilde{B}$,
$\delta_{q}$	the weight of ${\tilde{\alpha }}_{q}$,
Ω	set of PF number,
$Y=\left\{Y_{1},Y_{2},\cdots ,Y_{m}\right\}$	set of scheme,
$C=\left\{C_{1},C_{2},\cdots ,C_{n}\right\}$	set of criteria,
$E=\left\{E_{1},E_{2},\cdots ,E_{r}\right\}$	set of expert,
${\tilde{\Theta }}^{\left(g\right)}$	assessment matrix of *g*th expert,
$\nu_{g}$	the weight of *g*th expert,
$\tilde{\Theta }={\left({\tilde{h}}_{pq}\right)}_{m\times n}$	PF group assessment matrix,
$N_{q}$	average performance value of all schemes for every criteria,
$\varrho_{q}$	variation value,
$\delta_{q}^{o}$	objective weight of criteria,
$\delta_{q}^{s}$	subjective weight of criteria,
$h_{q}^{NIS}$	negative ideal solutions,
$h_{q}^{PIS}$	positive ideal solutions,
${ℵ}_{NIS}^{\left(1\right)}$	appraisal score of negative ideal solutions by PFGWA operator,
${ℵ}_{p}^{\left(1\right)}$	appraisal score of *p*th scheme by PFGWA operator,
${ℵ}_{PIS}^{\left(1\right)}$	appraisal score of positive ideal solutions by PFGWA operator,
$\vartheta_{p}^{\left(1\right)},\vartheta_{p}^{\left(2\right)}$	closeness grade,
$K_{p}^{\left(1\right)}$, $K_{p}^{\left(2\right)}$, $K_{p}^{\left(3\right)}$	the relative importance of *p*th scheme,
$K_{p}\left(p=1\left(1\right)m\right)$	synthetic utility value of *p*th scheme.

## Appendix A. The Proof of Theorem 1

**Proof.** (P1) and (P2) can be proved directly, but it is omitted here. (P3): It is supposed that $\tilde{A}$ and $\tilde{B}$ are two PF sets. If $\tilde{A}=\tilde{B}$, then we have ${\tilde{\mu }}_{\tilde{A}}\left(x_{q}\right)={\tilde{\mu }}_{\tilde{B}}\left(x_{q}\right)$, ${\tilde{\eta }}_{\tilde{A}}\left(x_{q}\right)={\tilde{\eta }}_{\tilde{B}}\left(x_{q}\right)$ and ${\tilde{\xi }}_{\tilde{A}}\left(x_{q}\right)={\tilde{\xi }}_{\tilde{B}}\left(x_{q}\right)$. Accordingly, based on the Equation (11), we can attain that $SM\left(\tilde{A},\tilde{B}\right)=1$ holds. Further, If $SM\left(\tilde{A},\tilde{B}\right)=1$, then, by means of Equation (11), we have

(A1) $$\begin{array}{cc} \; & \sum_{q=1}^{n}\left[\min\left\{{\tilde{\mu }}_{\tilde{A}}\left(x_{q}\right),{\tilde{\mu }}_{\tilde{B}}\left(x_{q}\right)\right\}+\min\left\{\left(1-{\tilde{\eta }}_{\tilde{A}}\left(x_{q}\right)\right),\left(1-{\tilde{\eta }}_{\tilde{B}}\left(x_{q}\right)\right)\right\}+\min\left\{\left(1-{\tilde{\xi }}_{\tilde{A}}\left(x_{q}\right)\right),\left(1-{\tilde{\xi }}_{\tilde{B}}\left(x_{q}\right)\right)\right\}\right]\\ = & \sum_{q=1}^{n}\left[\max\left\{{\tilde{\mu }}_{\tilde{A}}\left(x_{q}\right),{\tilde{\mu }}_{\tilde{B}}\left(x_{q}\right)\right\}+\max\left\{\left(1-{\tilde{\eta }}_{\tilde{A}}\left(x_{q}\right)\right),\left(1-{\tilde{\eta }}_{\tilde{B}}\left(x_{q}\right)\right)\right\}+\max\left\{\left(1-{\tilde{\xi }}_{\tilde{A}}\left(x_{q}\right)\right),\left(1-{\tilde{\xi }}_{\tilde{B}}\left(x_{q}\right)\right)\right\}\right]. \end{array}$$

Because $\min\left\{{\tilde{\mu }}_{\tilde{A}}\left(x_{q}\right),{\tilde{\mu }}_{\tilde{B}}\left(x_{q}\right)\right\}=\max\left\{{\tilde{\mu }}_{\tilde{A}}\left(x_{q}\right),{\tilde{\mu }}_{\tilde{B}}\left(x_{q}\right)\right\}$, $\min\{\left(1-{\tilde{\eta }}_{\tilde{A}}\left(x_{q}\right)\right),$$\left(1-{\tilde{\eta }}_{\tilde{B}}\left(x_{q}\right)\right)\}=\max\left\{\left(1-{\tilde{\eta }}_{\tilde{A}}\left(x_{q}\right)\right),\left(1-{\tilde{\eta }}_{\tilde{B}}\left(x_{q}\right)\right)\right\}$ and $\min\left\{\left(1-{\tilde{\xi }}_{\tilde{A}}\left(x_{q}\right)\right),\left(1-{\tilde{\xi }}_{\tilde{B}}\left(x_{q}\right)\right)\right\}=\max\{\left(1-{\tilde{\xi }}_{\tilde{A}}\left(x_{q}\right)\right),$$\left(1-{\tilde{\xi }}_{\tilde{B}}\left(x_{q}\right))\right\}$ hold. Hence, when Equation (46) holds, we can attain that ${\tilde{\mu }}_{\tilde{A}}\left(x_{q}\right),{\tilde{\mu }}_{\tilde{B}}\left(x_{q}\right)$, $\left(1-{\tilde{\eta }}_{\tilde{A}}\left(x_{q}\right)\right),\left(1-{\tilde{\eta }}_{\tilde{B}}\left(x_{q}\right)\right)$, and $\left(1-{\tilde{\xi }}_{\tilde{A}}\left(x_{q}\right)\right),\left(1-{\tilde{\xi }}_{\tilde{B}}\left(x_{q}\right)\right)$ are true. Thus, we have $\tilde{A}=\tilde{B}$ (P4): It is assumed that $\tilde{A}$, $\tilde{B}$, and $\tilde{C}$ are three PF sets. If $\tilde{A}\subseteq \tilde{B}\subseteq \tilde{A}$, then one has ${\tilde{\mu }}_{\tilde{A}}\left(x_{q}\right)\leq {\tilde{\mu }}_{\tilde{B}}\left(x_{q}\right)\leq {\tilde{\mu }}_{\tilde{C}}\left(x_{q}\right)$, ${\tilde{\eta }}_{\tilde{A}}\left(x_{q}\right)\geq {\tilde{\eta }}_{\tilde{B}}\left(x_{q}\right)\geq {\tilde{\eta }}_{\tilde{C}}\left(x_{q}\right)$, and ${\tilde{\xi }}_{\tilde{A}}\left(x_{q}\right)\geq {\tilde{\xi }}_{\tilde{B}}\left(x_{q}\right)\geq {\tilde{\xi }}_{\tilde{C}}\left(x_{q}\right)$. Further, $\left(1-{\tilde{\eta }}_{\tilde{A}}\left(x_{q}\right)\right)\leq \left(1-{\tilde{\eta }}_{\tilde{B}}\left(x_{q}\right)\right)\leq \left(1-{\tilde{\eta }}_{\tilde{C}}\left(x_{q}\right)\right)$ and $\left(1-{\tilde{\xi }}_{\tilde{A}}\left(x_{q}\right)\right)\leq \left(1-{\tilde{\xi }}_{\tilde{B}}\left(x_{q}\right)\right)\leq \left(1-{\tilde{\xi }}_{\tilde{C}}\left(x_{q}\right)\right)$. Therefore,

$$\begin{array}{c} SM\left(\tilde{A},\tilde{C}\right)=\frac{\sum_{q=1}^{n}\left[\min\left\{{\tilde{\mu }}_{\tilde{A}}\left(x_{q}\right),{\tilde{\mu }}_{\tilde{C}}\left(x_{q}\right)\right\}+\min\left\{\left(1-{\tilde{\eta }}_{\tilde{A}}\left(x_{q}\right)\right),\left(1-{\tilde{\eta }}_{\tilde{C}}\left(x_{q}\right)\right)\right\}+\min\left\{\left(1-{\tilde{\xi }}_{\tilde{A}}\left(x_{q}\right)\right),\left(1-{\tilde{\xi }}_{\tilde{C}}\left(x_{q}\right)\right)\right\}\right]}{\sum_{q=1}^{n}\left[\max\left\{{\tilde{\mu }}_{\tilde{A}}\left(x_{q}\right),{\tilde{\mu }}_{\tilde{C}}\left(x_{q}\right)\right\}+\max\left\{\left(1-{\tilde{\eta }}_{\tilde{A}}\left(x_{q}\right)\right),\left(1-{\tilde{\eta }}_{\tilde{C}}\left(x_{q}\right)\right)\right\}+\max\left\{\left(1-{\tilde{\xi }}_{\tilde{A}}\left(x_{q}\right)\right),\left(1-{\tilde{\xi }}_{\tilde{C}}\left(x_{q}\right)\right)\right\}\right]}. \end{array}$$

(A2) $$\begin{array}{c} SM\left(\tilde{A},\tilde{C}\right)=\frac{\sum_{q=1}^{n}\left[{\tilde{\mu }}_{\tilde{A}}\left(x_{q}\right)+\left(1-{\tilde{\eta }}_{\tilde{A}}\left(x_{q}\right)\right)+\left(1-{\tilde{\xi }}_{\tilde{A}}\left(x_{q}\right)\right)\right]}{\sum_{q=1}^{n}\left[{\tilde{\mu }}_{\tilde{C}}\left(x_{q}\right)+\left(1-{\tilde{\eta }}_{\tilde{C}}\left(x_{q}\right)\right)+\left(1-{\tilde{\xi }}_{\tilde{C}}\left(x_{q}\right)\right)\right]}. \end{array}$$

Further,

(A3) $$\begin{array}{c} SM\left(\tilde{A},\tilde{B}\right)=\frac{\sum_{q=1}^{n}\left[{\tilde{\mu }}_{\tilde{A}}\left(x_{q}\right)+\left(1-{\tilde{\eta }}_{\tilde{A}}\left(x_{q}\right)\right)+\left(1-{\tilde{\xi }}_{\tilde{A}}\left(x_{q}\right)\right)\right]}{\sum_{q=1}^{n}\left[{\tilde{\mu }}_{\tilde{B}}\left(x_{q}\right)+\left(1-{\tilde{\eta }}_{\tilde{B}}\left(x_{q}\right)\right)+\left(1-{\tilde{\xi }}_{\tilde{B}}\left(x_{q}\right)\right)\right]}. \end{array}$$

With the aid of Equations (46)–(48), we can acquire $SM\left(\tilde{A},\tilde{B}\right)\geq SM\left(\tilde{A},\tilde{C}\right)$. By the same manner, we have $SM\left(\tilde{A},\tilde{C}\right)\geq SM\left(\tilde{B},\tilde{C}\right)$. □

## Appendix B. The Proof of Theorem 3

**Proof.** We can prove it with the help of mathematical induction on *n*. For n=2, one has

$$\begin{array}{c} PFGDWA\left({\tilde{\alpha }}_{1},{\tilde{\alpha }}_{2}\right)=\delta_{1}{\tilde{\alpha }}_{1}\tilde{⊕}\delta_{2}{\tilde{\alpha }}_{2}, \end{array}$$

because $\delta_{1}{\tilde{\alpha }}_{1}$ and $\delta_{2}{\tilde{\alpha }}_{2}$ are PF numbers. Based on Definition 8, their integrated value $\delta_{1}{\tilde{\alpha }}_{1}⊕\delta_{2}{\tilde{\alpha }}_{2}$ is also a PF number, and we have

$$\begin{array}{cc} \; & PFGDWA\left({\tilde{\alpha }}_{1},{\tilde{\alpha }}_{2}\right)=\delta_{1}{\tilde{\alpha }}_{1}\tilde{⊕}\delta_{2}{\tilde{\alpha }}_{2}\\ \; & =\left({\left(1+{\left(\frac{1}{t}\left({\left(\Phi_{t}^{s}\left({\tilde{\mu }}_{{\tilde{\alpha }}_{1}}\right)\right)}^{\delta_{1}}-1\right)\right)}^{-\frac{1}{s}}\right)}^{-1},{\left(1+{\left(\frac{1}{t}\left({\left(\Psi_{t}^{s}\left({\tilde{\eta }}_{{\tilde{\alpha }}_{1}}\right)\right)}^{\delta_{1}}-1\right)\right)}^{\frac{1}{s}}\right)}^{-1},{\left(1+{\left(\frac{1}{t}\left({\left(\Psi_{t}^{s}\left({\tilde{\xi }}_{{\tilde{\alpha }}_{1}}\right)\right)}^{\delta_{1}}-1\right)\right)}^{\frac{1}{s}}\right)}^{-1}\right)\\ \; & \tilde{⊕}\left({\left(1+{\left(\frac{1}{t}\left({\left(\Phi_{t}^{s}\left({\tilde{\mu }}_{{\tilde{\alpha }}_{2}}\right)\right)}^{\delta_{2}}-1\right)\right)}^{-\frac{1}{s}}\right)}^{-1},{\left(1+{\left(\frac{1}{t}\left({\left(\Psi_{t}^{s}\left({\tilde{\eta }}_{{\tilde{\alpha }}_{2}}\right)\right)}^{\delta_{2}}-1\right)\right)}^{\frac{1}{s}}\right)}^{-1},{\left(1+{\left(\frac{1}{t}\left({\left(\Psi_{t}^{s}\left({\tilde{\xi }}_{{\tilde{\alpha }}_{2}}\right)\right)}^{\delta_{2}}-1\right)\right)}^{\frac{1}{s}}\right)}^{-1}\right)\\ \; & =\left({\left(1+{\left(\frac{1}{t}\left(\prod_{q=1}^{2}{\left(\Phi_{t}^{s}\left({\tilde{\mu }}_{{\tilde{\alpha }}_{q}}\right)\right)}^{\delta_{q}}-1\right)\right)}^{-\frac{1}{s}}\right)}^{-1},{\left(1+{\left(\frac{1}{t}\left(\prod_{q=1}^{2}{\left(\Psi_{t}^{s}\left({\tilde{\eta }}_{{\tilde{\alpha }}_{q}}\right)\right)}^{\delta_{q}}-1\right)\right)}^{\frac{1}{s}}\right)}^{-1},{\left(1+{\left(\frac{1}{t}\left(\prod_{q=1}^{2}{\left(\Psi_{t}^{s}\left({\tilde{\xi }}_{{\tilde{\alpha }}_{q}}\right)\right)}^{\delta_{q}}-1\right)\right)}^{\frac{1}{s}}\right)}^{-1}\right). \end{array}$$

Therefore, Equation (17) keeps for n=2. It is assumed that Equation (17) holds for n=d, namely,

$$\begin{array}{cc} \; & PFGDWA\left({\tilde{\alpha }}_{1},{\tilde{\alpha }}_{2},\cdots ,{\tilde{\alpha }}_{n}\right)=\overset{d}{\underset{q=1}{\tilde{⊕}}}\left(\delta_{q}{\tilde{\alpha }}_{q}\right)\\ \; & \left({\left(1+{\left(\frac{1}{t}\left(\prod_{q=1}^{d}{\left(\Phi_{t}^{s}\left({\tilde{\mu }}_{{\tilde{\alpha }}_{q}}\right)\right)}^{\delta_{q}}-1\right)\right)}^{-\frac{1}{s}}\right)}^{-1},{\left(1+{\left(\frac{1}{t}\left(\prod_{q=1}^{d}{\left(\Psi_{t}^{s}\left({\tilde{\eta }}_{{\tilde{\alpha }}_{q}}\right)\right)}^{\delta_{q}}-1\right)\right)}^{\frac{1}{s}}\right)}^{-1},{\left(1+{\left(\frac{1}{t}\left(\prod_{q=1}^{d}{\left(\Psi_{t}^{s}\left({\tilde{\xi }}_{{\tilde{\alpha }}_{q}}\right)\right)}^{\delta_{q}}-1\right)\right)}^{\frac{1}{s}}\right)}^{-1}\right). \end{array}$$

Then, when n=d+1, based on the operations in Definition 8, one has

$$\begin{array}{cc} \; & PFGDWA\left({\tilde{\alpha }}_{1},{\tilde{\alpha }}_{2},\cdots ,{\tilde{\alpha }}_{d},{\tilde{\alpha }}_{d+1}\right)=\overset{d+1}{\underset{q=1}{\tilde{⊕}}}\left(\delta_{q}{\tilde{\alpha }}_{q}\right)=\delta_{1}{\tilde{\alpha }}_{1}\tilde{⊕}\delta_{2}{\tilde{\alpha }}_{2}\tilde{⊕}\cdots \tilde{⊕}\delta_{n}{\tilde{\alpha }}_{n}\tilde{⊕}\delta_{d+1}{\tilde{\alpha }}_{d+1}\\ \; & =\left({\left(1+{\left(\frac{1}{t}\left(\prod_{q=1}^{d}{\left(\Phi_{t}^{s}\left({\tilde{\mu }}_{{\tilde{\alpha }}_{q}}\right)\right)}^{\delta_{q}}-1\right)\right)}^{-\frac{1}{s}}\right)}^{-1},{\left(1+{\left(\frac{1}{t}\left(\prod_{q=1}^{d}{\left(\Psi_{t}^{s}\left({\tilde{\eta }}_{{\tilde{\alpha }}_{q}}\right)\right)}^{\delta_{q}}-1\right)\right)}^{\frac{1}{s}}\right)}^{-1},{\left(1+{\left(\frac{1}{t}\left(\prod_{q=1}^{d}{\left(\Psi_{t}^{s}\left({\tilde{\xi }}_{{\tilde{\alpha }}_{q}}\right)\right)}^{\delta_{q}}-1\right)\right)}^{\frac{1}{s}}\right)}^{-1}\right)\\ \; & \tilde{⊕}\left({\left(1+{\left(\frac{1}{t}\left({\left(\Phi_{t}^{s}\left({\tilde{\mu }}_{{\tilde{\alpha }}_{q}}\right)\right)}^{\delta_{d+1}}-1\right)\right)}^{-\frac{1}{s}}\right)}^{-1},{\left(1+{\left(\frac{1}{t}\left({\left(\Psi_{t}^{s}\left({\tilde{\eta }}_{{\tilde{\alpha }}_{q}}\right)\right)}^{\delta_{d+1}}-1\right)\right)}^{\frac{1}{s}}\right)}^{-1},{\left(1+{\left(\frac{1}{t}\left({\left(\Psi_{t}^{s}\left({\tilde{\xi }}_{{\tilde{\alpha }}_{q}}\right)\right)}^{\delta_{d+1}}-1\right)\right)}^{\frac{1}{s}}\right)}^{-1}\right)\\ \; & =\left({\left(1+{\left(\frac{1}{t}\left(\prod_{q=1}^{d+1}{\left(\Phi_{t}^{s}\left({\tilde{\mu }}_{{\tilde{\alpha }}_{q}}\right)\right)}^{\delta_{q}}-1\right)\right)}^{-\frac{1}{s}}\right)}^{-1},{\left(1+{\left(\frac{1}{t}\left(\prod_{q=1}^{d+1}{\left(\Psi_{t}^{s}\left({\tilde{\eta }}_{{\tilde{\alpha }}_{q}}\right)\right)}^{\delta_{q}}-1\right)\right)}^{\frac{1}{s}}\right)}^{-1},{\left(1+{\left(\frac{1}{t}\left(\prod_{q=1}^{d+1}{\left(\Psi_{t}^{s}\left({\tilde{\xi }}_{{\tilde{\alpha }}_{q}}\right)\right)}^{\delta_{q}}-1\right)\right)}^{\frac{1}{s}}\right)}^{-1}\right). \end{array}$$

Based on the above steps, we can attain that $PFGDWA\left({\tilde{\alpha }}_{1},{\tilde{\alpha }}_{2},\cdots ,{\tilde{\alpha }}_{d},{\tilde{\alpha }}_{d+1}\right)$ is a PF number, and Equation (17) holds for n=d+1. Hence, Equation (17) keeps for all *n*. Accordingly, the theorem is proved. □

## Appendix C. The Proof of Property 1

**Proof.** Since ${\tilde{\alpha }}_{q}=\tilde{\alpha }=\left({\tilde{\mu }}_{\tilde{\alpha }},{\tilde{\eta }}_{\tilde{\alpha }},{\tilde{\xi }}_{\tilde{\alpha }}\right)$ for all *q*, then one has

$$\begin{array}{cc} \; & PFGDWA\left({\tilde{\alpha }}_{1},{\tilde{\alpha }}_{2},\cdots ,{\tilde{\alpha }}_{n}\right)=\overset{n}{\underset{q=1}{\tilde{⊕}}}\left(\delta_{q}{\tilde{\alpha }}_{q}\right)\\ \; & =\left({\left(1+{\left(\frac{1}{t}\left(\prod_{q=1}^{n}{\left(\Phi_{t}^{s}\left({\tilde{\mu }}_{{\tilde{\alpha }}_{q}}\right)\right)}^{\delta_{q}}-1\right)\right)}^{-\frac{1}{s}}\right)}^{-1},{\left(1+{\left(\frac{1}{t}\left(\prod_{q=1}^{n}{\left(\Psi_{t}^{s}\left({\tilde{\eta }}_{{\tilde{\alpha }}_{q}}\right)\right)}^{\delta_{q}}-1\right)\right)}^{\frac{1}{s}}\right)}^{-1},{\left(1+{\left(\frac{1}{t}\left(\prod_{q=1}^{n}{\left(\Psi_{t}^{s}\left({\tilde{\xi }}_{{\tilde{\alpha }}_{q}}\right)\right)}^{\delta_{q}}-1\right)\right)}^{\frac{1}{s}}\right)}^{-1}\right)\\ \; & =\left({\left(1+{\left(\frac{1}{t}\left(\prod_{q=1}^{n}{\left(\Phi_{t}^{s}\left({\tilde{\mu }}_{\tilde{\alpha }}\right)\right)}^{\delta_{q}}-1\right)\right)}^{-\frac{1}{s}}\right)}^{-1},{\left(1+{\left(\frac{1}{t}\left(\prod_{q=1}^{n}{\left(\Psi_{t}^{s}\left({\tilde{\eta }}_{\tilde{\alpha }}\right)\right)}^{\delta_{q}}-1\right)\right)}^{\frac{1}{s}}\right)}^{-1},{\left(1+{\left(\frac{1}{t}\left(\prod_{q=1}^{n}{\left(\Psi_{t}^{s}\left({\tilde{\xi }}_{\tilde{\alpha }}\right)\right)}^{\delta_{q}}-1\right)\right)}^{\frac{1}{s}}\right)}^{-1}\right)\\ \; & =\left({\left(1+{\left(\frac{1}{t}\left(\left(1+t{\left(\frac{{\tilde{\mu }}_{\tilde{\alpha }}}{1-{\tilde{\mu }}_{\tilde{\alpha }}}\right)}^{s}\right)-1\right)\right)}^{-\frac{1}{s}}\right)}^{-1},{\left(1+{\left(\frac{1}{t}\left(\left(1+t{\left(\frac{1-{\tilde{\eta }}_{\tilde{\alpha }}}{{\tilde{\eta }}_{\tilde{\alpha }}}\right)}^{s}\right)-1\right)\right)}^{\frac{1}{s}}\right)}^{-1},{\left(1+{\left(\frac{1}{t}\left(\left(1+t{\left(\frac{1-{\tilde{\xi }}_{\tilde{\alpha }}}{{\tilde{\xi }}_{\tilde{\alpha }}}\right)}^{s}\right)-1\right)\right)}^{\frac{1}{s}}\right)}^{-1}\right)\\ \; & =\left({\left(1+{\left(\frac{{\tilde{\mu }}_{\tilde{\alpha }}}{1-{\tilde{\mu }}_{\tilde{\alpha }}}\right)}^{-1}\right)}^{-1},{\left(1+\frac{1-{\tilde{\eta }}_{\tilde{\alpha }}}{{\tilde{\eta }}_{\tilde{\alpha }}}\right)}^{-1},{\left(1+\frac{1-{\tilde{\xi }}_{\tilde{\alpha }}}{{\tilde{\xi }}_{\tilde{\alpha }}}\right)}^{-1}\right)=\left({\tilde{\mu }}_{\tilde{\alpha }},{\tilde{\eta }}_{\tilde{\alpha }},{\tilde{\xi }}_{\tilde{\alpha }}\right). \end{array}$$

Hence, $PFGDWA\left({\tilde{\alpha }}_{1},{\tilde{\alpha }}_{2},\cdots ,{\tilde{\alpha }}_{n}\right)=\tilde{\alpha }$ holds. □

## Appendix D. The Proof of Property 2

**Proof.** Since ${\tilde{\mu }}_{{\tilde{\alpha }}_{q}}\geq {\tilde{\tilde{\eta }}}_{{\tilde{\tilde{\alpha }}}_{q}}$ holds, then

$$\begin{array}{cc} \; & \;{\left(\Phi_{t}^{s}\left({\tilde{\mu }}_{{\tilde{\alpha }}_{q}}\right)\right)}^{\delta_{q}}\geq {\left(\Phi_{t}^{s}\left({\tilde{\mu }}_{{\tilde{\tilde{\alpha }}}_{q}}\right)\right)}^{\delta_{q}}\\ \; & \Rightarrow \frac{1}{t}\left(\prod_{q=1}^{n}{\left(\Phi_{t}^{s}\left({\tilde{\mu }}_{{\tilde{\alpha }}_{q}}\right)\right)}^{\delta_{q}}-1\right)\geq \frac{1}{t}\left(\prod_{q=1}^{n}{\left(\Phi_{t}^{s}\left({\tilde{\tilde{\mu }}}_{{\tilde{\tilde{\alpha }}}_{q}}\right)\right)}^{\delta_{q}}-1\right)\\ \; & \Rightarrow 1+{\left(\frac{1}{t}\left(\prod_{q=1}^{n}{\left(\Phi_{t}^{s}\left({\tilde{\mu }}_{{\tilde{\alpha }}_{q}}\right)\right)}^{\delta_{q}}-1\right)\right)}^{-\frac{1}{s}}\leq 1+{\left(\frac{1}{t}\left(\prod_{q=1}^{n}{\left(\Phi_{t}^{s}\left({\tilde{\tilde{\mu }}}_{{\tilde{\tilde{\alpha }}}_{q}}\right)\right)}^{\delta_{q}}-1\right)\right)}^{-\frac{1}{s}}\\ \; & \Rightarrow {\left(1+{\left(\frac{1}{t}\left(\prod_{q=1}^{n}{\left(\Phi_{t}^{s}\left({\tilde{\mu }}_{{\tilde{\alpha }}_{q}}\right)\right)}^{\delta_{q}}-1\right)\right)}^{-\frac{1}{s}}\right)}^{-1}\geq {\left(1+{\left(\frac{1}{t}\left(\prod_{q=1}^{n}{\left(\Phi_{t}^{s}\left({\tilde{\tilde{\mu }}}_{{\tilde{\tilde{\alpha }}}_{q}}\right)\right)}^{\delta_{q}}-1\right)\right)}^{-\frac{1}{s}}\right)}^{-1}. \end{array}$$

By the same manner, because ${\tilde{\eta }}_{{\tilde{\alpha }}_{q}}\leq {\tilde{\tilde{\eta }}}_{{\tilde{\tilde{\alpha }}}_{q}}$ and ${\tilde{\xi }}_{{\tilde{\alpha }}_{q}}\leq {\tilde{\tilde{\xi }}}_{{\tilde{\tilde{\alpha }}}_{q}}$, we have

$$\begin{array}{cc} \; & {\left(1+{\left(\frac{1}{t}\left(\prod_{q=1}^{n}{\left(\Psi_{t}^{s}\left({\tilde{\eta }}_{{\tilde{\alpha }}_{q}}\right)\right)}^{\delta_{q}}-1\right)\right)}^{\frac{1}{s}}\right)}^{-1}\leq {\left(1+{\left(\frac{1}{t}\left(\prod_{q=1}^{n}{\left(\Psi_{t}^{s}\left({\tilde{\tilde{\eta }}}_{{\tilde{\tilde{\alpha }}}_{q}}\right)\right)}^{\delta_{q}}-1\right)\right)}^{\frac{1}{s}}\right)}^{-1},\\ \; & {\left(1+{\left(\frac{1}{t}\left(\prod_{q=1}^{n}{\left(\Psi_{t}^{s}\left({\tilde{\xi }}_{{\tilde{\alpha }}_{q}}\right)\right)}^{\delta_{q}}-1\right)\right)}^{\frac{1}{s}}\right)}^{-1}\leq {\left(1+{\left(\frac{1}{t}\left(\prod_{q=1}^{n}{\left(\Psi_{t}^{s}\left({\tilde{\tilde{\xi }}}_{{\tilde{\tilde{\alpha }}}_{q}}\right)\right)}^{\delta_{q}}-1\right)\right)}^{\frac{1}{s}}\right)}^{-1}. \end{array}$$

Further, by the Definition 3, we have $PFGDWA\left({\tilde{\alpha }}_{1},{\tilde{\alpha }}_{2},\cdots ,{\tilde{\alpha }}_{n}\right)>PFGDWA\left({\tilde{\tilde{\alpha }}}_{1},{\tilde{\tilde{\alpha }}}_{2},\cdots ,{\tilde{\tilde{\alpha }}}_{n}\right)$. Additionally, when ${\tilde{\mu }}_{{\tilde{\alpha }}_{q}}={\tilde{\tilde{\eta }}}_{{\tilde{\tilde{\alpha }}}_{q}}$, ${\tilde{\eta }}_{{\tilde{\alpha }}_{q}}={\tilde{\tilde{\eta }}}_{{\tilde{\tilde{\alpha }}}_{q}}$, and ${\tilde{\xi }}_{{\tilde{\alpha }}_{q}}={\tilde{\tilde{\xi }}}_{{\tilde{\tilde{\alpha }}}_{q}}$, we have $PFGDWA\left({\tilde{\alpha }}_{1},{\tilde{\alpha }}_{2},\cdots ,{\tilde{\alpha }}_{n}\right)=PFGDWA\left({\tilde{\tilde{\alpha }}}_{1},{\tilde{\tilde{\alpha }}}_{2},\cdots ,{\tilde{\tilde{\alpha }}}_{n}\right)$. Thus, $PFGDWA\left({\tilde{\alpha }}_{1},{\tilde{\alpha }}_{2},\cdots ,{\tilde{\alpha }}_{n}\right)\geq PFGDWA\left({\tilde{\tilde{\alpha }}}_{1},{\tilde{\tilde{\alpha }}}_{2},\cdots ,{\tilde{\tilde{\alpha }}}_{n}\right)$ holds. □

## Appendix E. The Proof of Property 3

**Proof.** By means of Property 1 and 2, we have

$$\begin{array}{cc} \; & PFGDWA\left({\tilde{\alpha }}_{1},{\tilde{\alpha }}_{2},\cdots ,{\tilde{\alpha }}_{n}\right)\leq PFGDWA\left({\tilde{\alpha }}_{1}^{+},{\tilde{\alpha }}_{2}^{+},\cdots ,{\tilde{\alpha }}_{n}^{+}\right)={\tilde{\alpha }}^{+},\\ \; & PFGDWA\left({\tilde{\alpha }}_{1},{\tilde{\alpha }}_{2},\cdots ,{\tilde{\alpha }}_{n}\right)\geq PFGDWA\left({\tilde{\alpha }}_{1}^{-},{\tilde{\alpha }}_{2}^{-},\cdots ,{\tilde{\alpha }}_{n}^{-}\right)={\tilde{\alpha }}^{-}. \end{array}$$

Hence, ${\tilde{\alpha }}^{-}\leq PFGDWA\left({\tilde{\alpha }}_{1},{\tilde{\alpha }}_{2},\cdots ,{\tilde{\alpha }}_{n}\right)\leq {\tilde{\alpha }}^{+}$ holds. □
